# Spatial Molecular Heterogeneity on Biofunctionalized Particles Quantified by Three-Dimensional Single-Molecule DNA-PAINT

**DOI:** 10.1021/acs.langmuir.5c02403

**Published:** 2025-08-13

**Authors:** Wei Shan Tan, Arthur M. de Jong, Menno W. J. Prins

**Affiliations:** † Department of Biomedical Engineering, 3169 Eindhoven University of Technology, Eindhoven 5612 AZ, The Netherlands; ‡ Institute for Complex Molecular Systems (ICMS), 3169 Eindhoven University of Technology, Eindhoven 5612 AZ, The Netherlands; § Department of Applied Physics and Science Education, 3169 Eindhoven University of Technology, Eindhoven 5612 AZ, The Netherlands; ∥ Helia Biomonitoring, Eindhoven 5612 AR, The Netherlands

## Abstract

Quantifying and controlling the spatial molecular heterogeneity on biofunctionalized particles is essential for understanding and improving their functionality in bioscience applications. Here, we describe an analysis framework based on single-molecule localization microscopy that can quantitatively assess the spatial molecular properties of affinity molecules conjugated to particles. We performed 3D DNA-PAINT imaging on biofunctionalized particles and established analysis methods to correlate single-molecule data to the particle outer surfaces, count the number of conjugated molecules, and quantify the spatial distributions of the conjugated molecules. We show that imaging data combined with simulation-based molecular counting gives access to high densities of conjugated molecules and enables quantification of their spatial distributions. The analysis is exemplified for particles with a diameter of 1 μm functionalized with single-stranded DNA molecules via two bioconjugation methods, namely, streptavidin–biotin coupling and PLL-*g*-PEG-based click chemistry. The data reveal interparticle and intraparticle spatial heterogeneities that are dependent on the bioconjugation methods and conditions. With the analysis framework, 3D DNA-PAINT imaging becomes a versatile characterization technique to study biofunctionalized particles and guide future biofunctionalization strategies for a wide range of applications.

## Graphical abstract

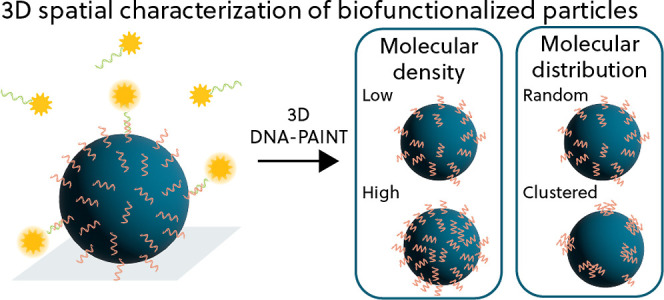

## Introduction

Biofunctionalized micro- and nanoparticles have broad applications in the biomedical field, ranging from the therapeutic delivery of drugs to diagnostic imaging and in vitro diagnostic testing.^1–3^ Typically, the surfaces of the particles are functionalized with affinity molecules to enable specific interactions with molecules, cells, and tissues. It is important to characterize and control the spatial properties of the conjugated biomolecules (number density, spatial distribution, etc.) in order to ensure the correct functionality and reproducibility of the particles. Several methods have been developed to quantify the average number of affinity molecules on particles, such as labeling the molecules with fluorescent dyes or radioactive isotopes.^4,5^ However, information about interparticle and intraparticle heterogeneity requires characterization techniques with very high spatial resolutions due to the small sizes and the high areal densities of the affinity molecules, typically 10^3^ to 10^5^ molecules per micrometer squared, corresponding to intermolecular distances in the range between 50 and 5 nm.

Single-molecule localization microscopy (SMLM) techniques present new avenues toward the quantification of biomolecules on surfaces with single-molecule resolution. By temporally separating fluorescent emissions of single fluorophores or fluorescent dyes, SMLM techniques avoid spatial overlaps between the point spread functions (PSFs) of individual fluorophores and, therefore, can determine the spatial coordinates of each fluorophore with high precision.^6^ DNA point accumulation in nanoscale topography (DNA-PAINT) is an SMLM technique based on the imaging of DNA probes with well-controlled event kinetics.^7,8^ Dye-labeled imager single-stranded DNA (ssDNA) transiently binds to complementary ssDNA (also known as docking strands) on the surface of interest, resulting in the temporal separation of fluorophores. In recent years, DNA-PAINT imaging has been used to quantify the number and orientation of biomolecules conjugated to nanoparticles and microparticles^9–11^ and to semiquantitatively describe the spatial distribution of biomolecules on spherical surfaces.^12,13^ In these studies, spatial distributions of biomolecules were described for relatively low conjugated biomolecule densities (<10^3^ molecules per micrometer squared)^12^ or for only a portion of the particle surfaces.^13^ Due to the limited statistics of the methods, a fully quantitative assessment of the spatial distribution of biomolecules on densely functionalized particles has not yet been achieved.

In this article, we describe the development and application of an analysis framework based on 3D DNA-PAINT imaging with an astigmatic lens, designed to improve access to the spatial localization of coupled biomolecules in high-density systems. The analysis framework is applied to biofunctionalized particles with a diameter of 1 μm to obtain quantitative insights into the molecular density and spatial distribution of the coupled biomolecules. A simulation model is used to compensate for spatial under- and oversampling effects, which yields a molecular counting approach that is adaptable to varying experimental parameters. Using statistical tools for spatial point analysis, we count the number of conjugated biomolecules and quantitatively describe the spatial distributions of these biomolecules. The analysis is demonstrated for ssDNA-functionalized particles that were prepared with two different bioconjugation methods, namely, streptavidin–biotin coupling and poly­(l-lysine)-grafted-poly­(ethylene glycol) (PLL-*g*-PEG)-based click coupling. The study reveals unexpected interparticle and intraparticle spatial heterogeneities that are dependent on the bioconjugation method and conditions.

## Results and Discussion

Figure 1 sketches the goals and approaches of the study. DNA-PAINT is an SMLM technique that relies on sequence-dependent DNA interactions to achieve super-resolution imaging. To achieve three-dimensional imaging, the astigmatism method was used, where a cylindrical lens is placed in the imaging path to laterally distort PSFs in the x and y directions related to the z position of the emitter,^14^ as shown in Figure 1B. In other words, typical 2D symmetrical PSFs are optically engineered to encode axial information. Calibrations of the astigmatism lens are used for decoding the distorted PSFs into z-position information; see Figure S8. In this study, we opted to use highly inclined and laminated optical sheet (HILO) imaging instead of the more-commonly used total internal reflection fluorescence (TIRF) setup. TIRF imaging enables a high signal-to-noise ratio of single-molecule imaging data but is limited to fluorescent events near the surface of the glass coverslip because the penetration depth is approximately a few hundred nanometers. HILO imaging overcomes this limitation by directing the illumination beam at a sharp angle through the sample.^15^ This allows for an imaging depth of up to 10 μm, albeit at the cost of a slightly lower signal-to-noise ratio. Since the particles have a mean diameter of 1 μm, HILO imaging enables studies of the whole particle surface.

**Figure 1:**
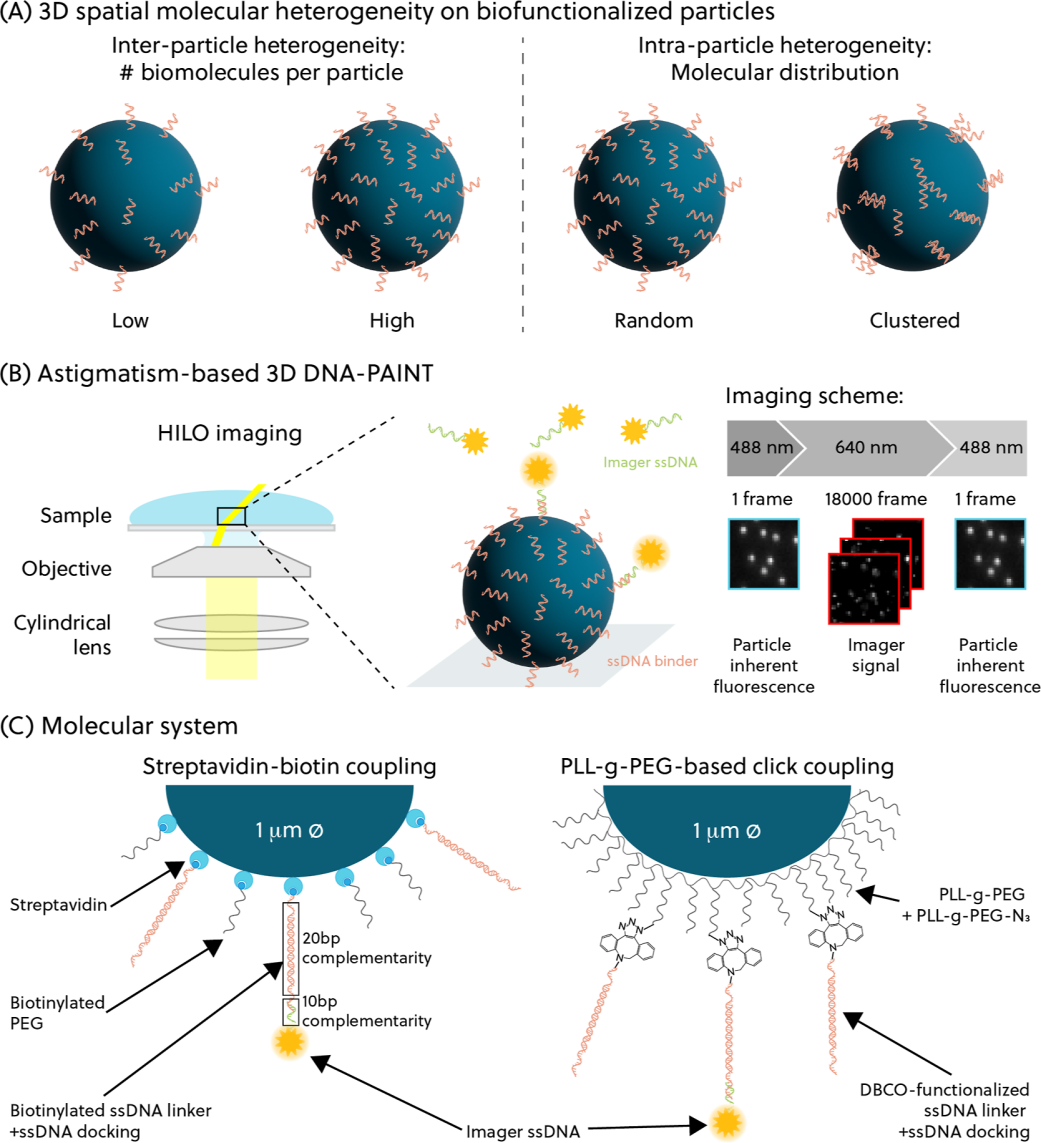
(A) Spatial molecular heterogeneity on biofunctionalized particles can be classified into two categories: (1) interparticle heterogeneity refers to the variability between particles in terms of the number of conjugated biomolecules. (2) Intraparticle heterogeneity refers to the spatial distribution of the conjugated biomolecule on each particle. (B) 3D DNA-PAINT is achieved by inserting a cylindrical lens, which introduces astigmatism on the PSF of the emitters, in the detection pathway. To image the entire particle, the HILO configuration was used. A varying imaging scheme was employed to obtain the particle locations and their respective DNA-PAINT localizations. (C) This analysis is demonstrated for two molecular systems. The particles were functionalized with ssDNA docking strands via streptavidin–biotin coupling or PLL-*g*-PEG-based click coupling.

The investigated ssDNA-functionalized particles in this article are commercially available magnetic particles with a diameter of 1 μm, coated with covalently attached streptavidin molecules or with negatively charged surface carboxylic acid groups. The particles were chosen for their wide range of applications such as for protein purification and particle-based sensors.^16,17^ The streptavidin-coated particles were functionalized with biotinylated, partially double-stranded DNA (dsDNA) molecules consisting of an ssDNA linker and an ssDNA docking strand that interacts with the imager strand. For the carboxylated particles, a low-fouling polymer layer, consisting of a mixture of PLL-*g*-PEG molecules and azide-functionalized PLL-*g*-PEG molecules (PLL-*g*-PEG-N_3_), was first coated onto the particle via electrostatic physisorption, followed by click-based coupling of the partially double-stranded DNA molecules to the PLL-*g*-PEG-coated particles. The coating of particles using PLL-*g*-PEG molecules is confirmed by zeta potential measurements, as shown in Figure S9. PLL-*g*-PEG-based systems are widely used for conjugating biomolecules to surfaces;^18–23^ thus, it is interesting to study how this system compares with the commercially available streptavidin-coated particles. The molecular systems used in this work are illustrated in Figure 1C.

Correlating the single-molecule data to the objects of interest (in this article, the particles) is an important feature for relating biofunctionality to physical locations on the particles. Figure 2A shows how we first identified the locations of the particles based on their raw fluorescence image (see Figure S2 for the localization uncertainties of the particles) and then drew a 2 μm by 2 μm region of interest (ROI) around the localized center of the particles. The particles used in this work are polymeric in nature and exhibit inherent fluorescence when illuminated with a 488 nm laser. The particle centers were determined by using ThunderSTORM,^24^ an ImageJ plugin, to localize features in the inherent fluorescence image. Two examples of particle ROIs are shown in Figure 2A. Figure 2B shows the number of localizations in particle ROIs for particles prepared in different conditions: (1) bare streptavidin-coated particles, (2) particles blocked with only PEG molecules, (3) particles functionalized with noncomplementary ssDNA docking strand and blocked with PEG molecules, and (4) particles functionalized with complementary ssDNA docking strands and blocked with PEG molecules. Conditions 1 to 3 serve as negative controls. The control experiments for the particles prepared via PLL-*g*-PEG-based click coupling are shown in Figure S10. By analyzing these particles, we found significant differences (p-value less than 0.001) between Condition 4 and the other conditions, thus giving confidence that the 3D DNA-PAINT localization data are related to the specific biofunctionality of the particles functionalized with complementary ssDNA docking strands.

**Figure 2:**
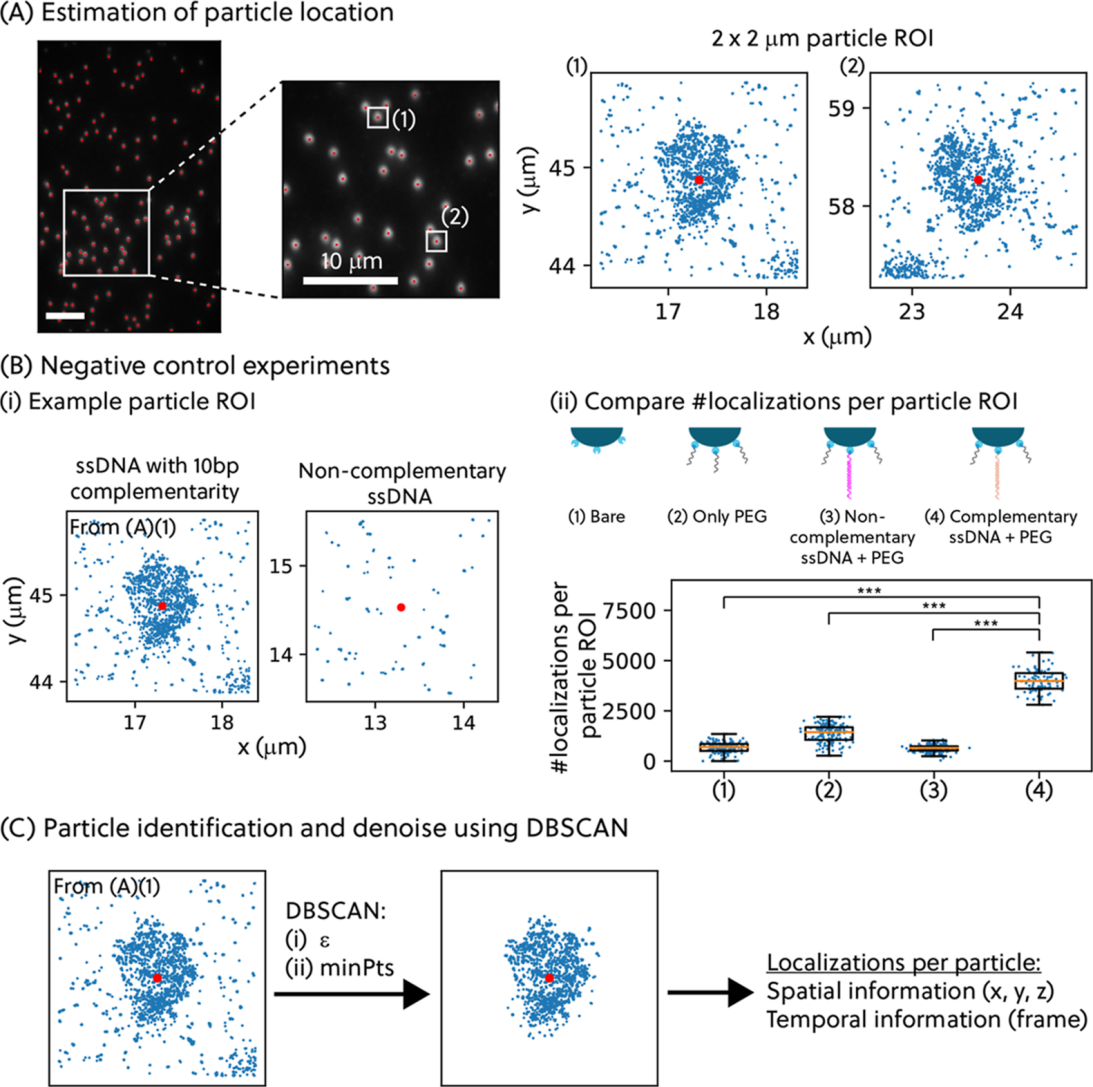
(A) The xy-locations of the particles were first estimated from images of the inherent fluorescence of the particles, when excited with the 488 nm laser. A 2 μm × 2 μm ROI (referred to as particle ROI) was drawn around each particle location in the DNA-PAINT localization data for further data processing. (B) The number of localizations per particle ROI was used to evaluate whether localizations observed within ROI are specific interactions or not. (i) Examples of particle ROIs. (ii) Number of localizations per particle ROI are plotted as boxplots for different cases. Box represents the 25 to 75% percentile, whiskers 5 to 95%. Each blue filled dot represents each particle ROI. *** indicates p-values less than 0.001. (C) A DBSCAN clustering algorithm was employed to obtain the localization data (*x*, *y*, *z*, and frame) that belongs to each biofunctionalized particle.

To differentiate the localizations belonging to a selected particle from the background (the glass surface), a clustering algorithm, known as density-based spatial clustering of applications with noise (DBSCAN), was applied to the localization data (specifically the xy-information on the localizations) in the particle ROI; see Figure 2C. Briefly, the DBSCAN algorithm employs a minimum density level estimation, based on a threshold for the number of points, minPts, within the radius ϵ with a to-be-chosen distance measure.^25,26^ The values for input parameters can be guided by the size of the particles and the expected number of localizations per particle. To ensure the reliability of the analysis, we empirically determined the input parameters by visually inspecting the clustering result. The particle localization cloud identification step is crucial for preventing errors in downstream analysis. Different sources of error in this analysis step are discussed in Section S2.1. Among established clustering algorithms, we chose DBSCAN for its capability to determine localization clouds of arbitrary shape and its good efficiency on large databases.^25^ The particles in our study have a spherical shape, but the use of DBSCAN ensures generalizability for studying particles of varying geometry. With this analysis step, we obtained the spatial information and temporal information on the localizations for each particle.

A first method to quantify the number of biomolecules on the particles is to directly count the number of binding events per particle: the direct counting (DC) approach. We interpret each binding event observed on the particle as a hybridization event between the imager strand and a unique ssDNA molecule on the particle. For this interpretation, it is important to know the average number of expected binding events per ssDNA docking molecule *n*_event_. For the conditions of the experiment in Figure 2, *n*_event_ was calculated to be 0.9, based on eq 1 and experimental parameters.

1 $$n_{event}=t_{meas}\times k_{on}\times c_{img}$$

where *t*_meas_ denotes measurement duration, *c*_img_ is the imager concentration, and *k*_on_ is the molecular association rate between ssDNA strands. The *k*_on_ parameter has been previously determined by performing a DNA-PAINT kinetic study on surfaces with well-defined single ssDNA docking strands on DNA origami and was found to be in the order of 10^6^ M^–1^ s^–1^.^7^ In this study, we assume a *k*_on_ reference value of 10^6^ M^–1^ s^–1^, which means that the data in this paper represent the origami-equivalent number of ssDNA molecules on the surface of the particle. This also means that the calculated *n*_event_ is the DNA-origami equivalent average number of binding events per ssDNA docking molecule. In the experiment, the average number of binding events per ssDNA docking molecule could be higher or lower than 0.9, depending on the true value of *k*_on_ in the experiment.

To count the number of binding events based on the localization data, we clustered the localizations based on time and position to obtain localizations per binding event. Briefly, localizations that were present in consecutive frames were considered binding event(s). As shown in Figure 3A, when the xy-information on these localizations are plotted, we observed multiple clouds of localization, indicating there were multiple binding events occurring within the observation frames. Based on this, we identified separate localization clouds (hence binding events) when the distance between the localization clouds was larger than the mean of the lateral localization uncertainty.

**Figure 3:**
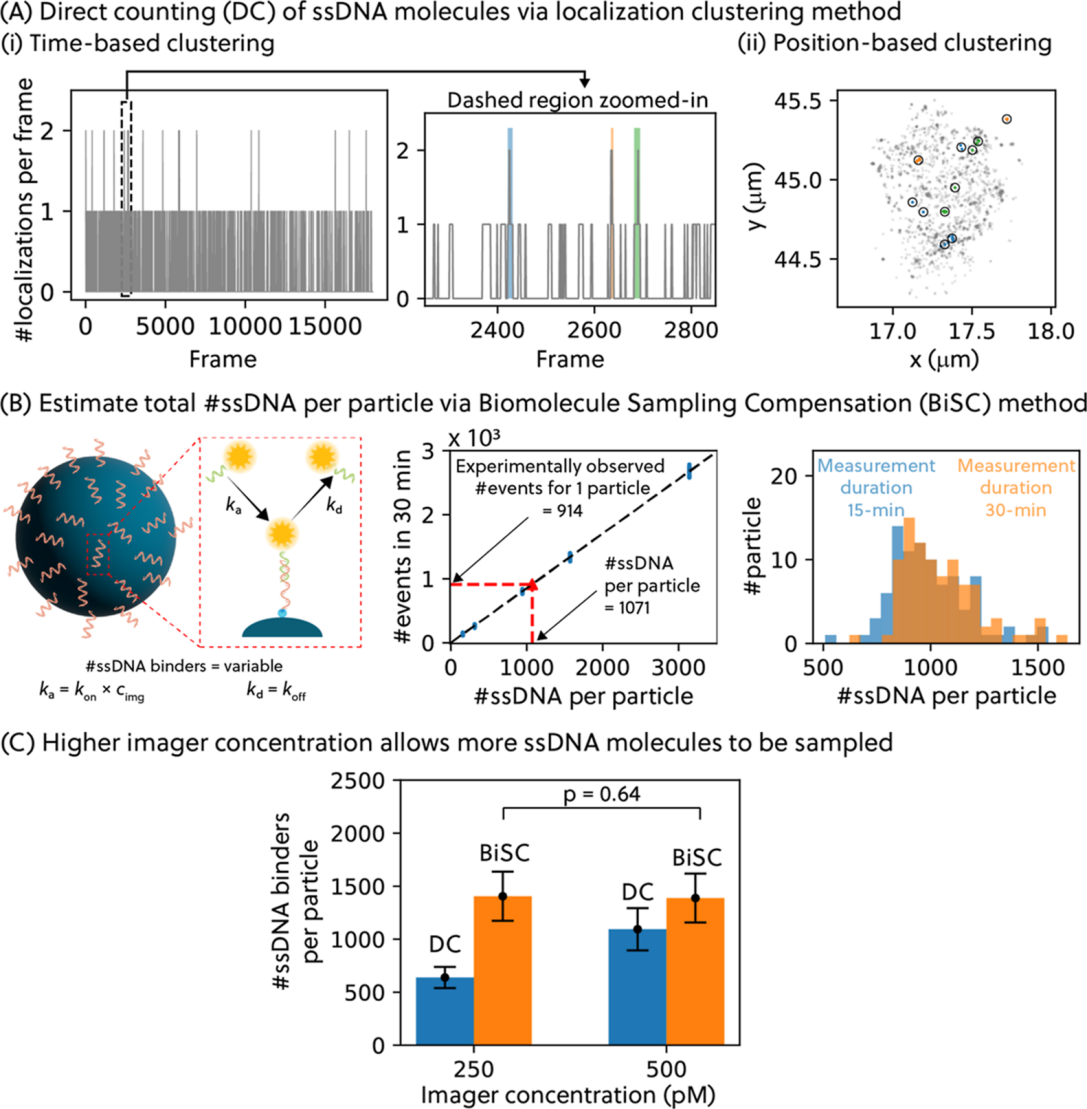
(A) The localization data per particle are clustered in time and space to obtain spatial information on each binding event that occurred. The localizations that occurred in consecutive frames were first identified (i), after which these localizations were clustered based on their xy-spatial locations (ii). The color of the localization data in (ii) corresponds to the respective frames they belong in (i). The data shown here is obtained from a representative particle measured in the experiment. (B) The Biomolecule Sampling Compensation (BiSC) method is a simulation-based approach that compensates for molecular undersampling. By simulating binding events for each docking strand, the method relates observed binding events to true molecular density and enables inverse estimation (left and middle panel). Moreover, BiSC quantification is independent of measurement duration (right panel). (C) For the same batch of biofunctionalized particles, more ssDNA molecules (more binding events) were observed when a higher imager concentration was used. However, when analyzed with the BiSC method, there is no significant difference in the number of ssDNA molecules per particle (*p* = 0.64) between the particles imaged with high or low imager concentration. Experimental data shown here are particles functionalized with 5 μM complementary ssDNA molecules using streptavidin–biotin coupling.

Due to the finite measurement time, we cannot guarantee that all ssDNA molecules present on the particles are probed by one or more imager strands. To compensate for potential under- or oversampling, an analysis was developed based on previous work,^27^ where we studied DNA-PAINT quantification of flat biofunctionalized surfaces. In the previous paper, we named the methodology Compensation for Binder Undersampling (CBiU). Here we propose to rename it Biomolecule Sampling Compensation (BiSC) because the new methodology compensates for undersampling as well as for oversampling; see Section S2.2. The methodology is summarized in Figure 3B. The analysis is based on a Monte Carlo model that simulates the hybridization events of imager strands to individual ssDNA docking strands based on known kinetic constants *k*_a_ and *k*_d_; see eq 2 and Section S2.2.

2 $$k_{a}=k_{on}\times c_{img},k_{d}=k_{off}$$

where *k*_off_ is the molecular dissociation rate constant of the ssDNA strands. *k*_off_ was quantified to be 1 s^–1^ in previous work.^27^

Using the simulation approach, the total number of observed binding events within the measurement duration is correlated to the total number of ssDNA molecules per particle. Using the relation found, we inversely estimated the number of ssDNA molecules per particle from the experimentally observed number of binding events per particle. Performing 3D DNA-PAINT imaging with higher imager concentration may result in multiple binding events per ssDNA docking strands (higher *n*_event_) and cause oversampling of molecules instead. By running the Monte Carlo simulation model for higher imager concentration, the BiSC methodology is equally capable of compensating for oversampling as well as undersampling of molecules.

Moreover, the BiSC analysis is preferred over the more well-known quantitative PAINT (qPAINT) approach. The qPAINT approach relies on the assumption that no double events occur during the measurement duration, i.e., only single binding events are observed in the region of interest at any time during the measurement.^28,29^ As seen in Figure 3A, this criterion is violated, thus rendering a qPAINT analysis unreliable for this study. In principle, one could adapt the imaging condition for reducing the number of double events, by reducing imager concentration and increasing the measurement duration to achieve the same statistics. However, it is impractical and undesirable to perform long measurements; therefore, a higher imager concentration was used in combination with the BiSC analysis. For precise quantification using the BiSC analysis, it is equally important to ensure that binding events within a small region of interest (i.e., within the particle ROI) can be reliably distinguished. In Section S2.2, we estimate that the maximum allowable emitter density per frame to avoid significant PSF overlap is approximately 3.0 μm^–2^. In our experimental data, the maximum number of localizations observed in any single frame for a given particle is two (see Section S3.11), which is well within this resolvable range, confirming the validity of the BiSC quantification under the chosen imaging conditions.

To verify the reliability of the methodology, we performed the BiSC analysis on data sets of different measurement durations (a full 30-min data set and a 15-min subset of the data set; see Figure 3B), showing that the quantification derived from the analysis is independent of the measurement duration. Moreover, when 3D DNA-PAINT measurements were performed on the same batch of biofunctionalized particles using different imager concentrations (Figure 3C), we observed an increase in the DC-quantified number of ssDNA molecules per particle, but the BiSC-quantified numbers showed no significant difference. Here, the DC-quantified number reflects the ssDNA docking molecules that were actually probed during the DNA-PAINT measurement, while the BiSC-quantified number estimates the total number of docking molecules present by compensating for molecular undersampling. This validates that the BiSC analysis is a reliable quantification approach for varying imaging conditions.

To analyze the spatial distribution of ssDNA molecules on the particles, we first averaged the spatial coordinates of the localizations belonging to one ssDNA molecule, obtaining the estimated spatial positions of the ssDNA molecules; see Figure 4A. The localizations belonging to one ssDNA molecule were obtained after performing the localization clustering step as described in the previous section. A common issue in astigmatism-based 3D SMLM techniques is that the axial localization uncertainty is typically worse than the lateral localization uncertainty (in the study of this article, one order of magnitude difference; see Figure S11). Due to the axial localization uncertainty, the observed positions of the ssDNA molecules may lie in or outside of the particles, which is physically not possible. In order to resolve this, we projected the ssDNA molecules onto the known spherical shape of the particle.

**Figure 4:**
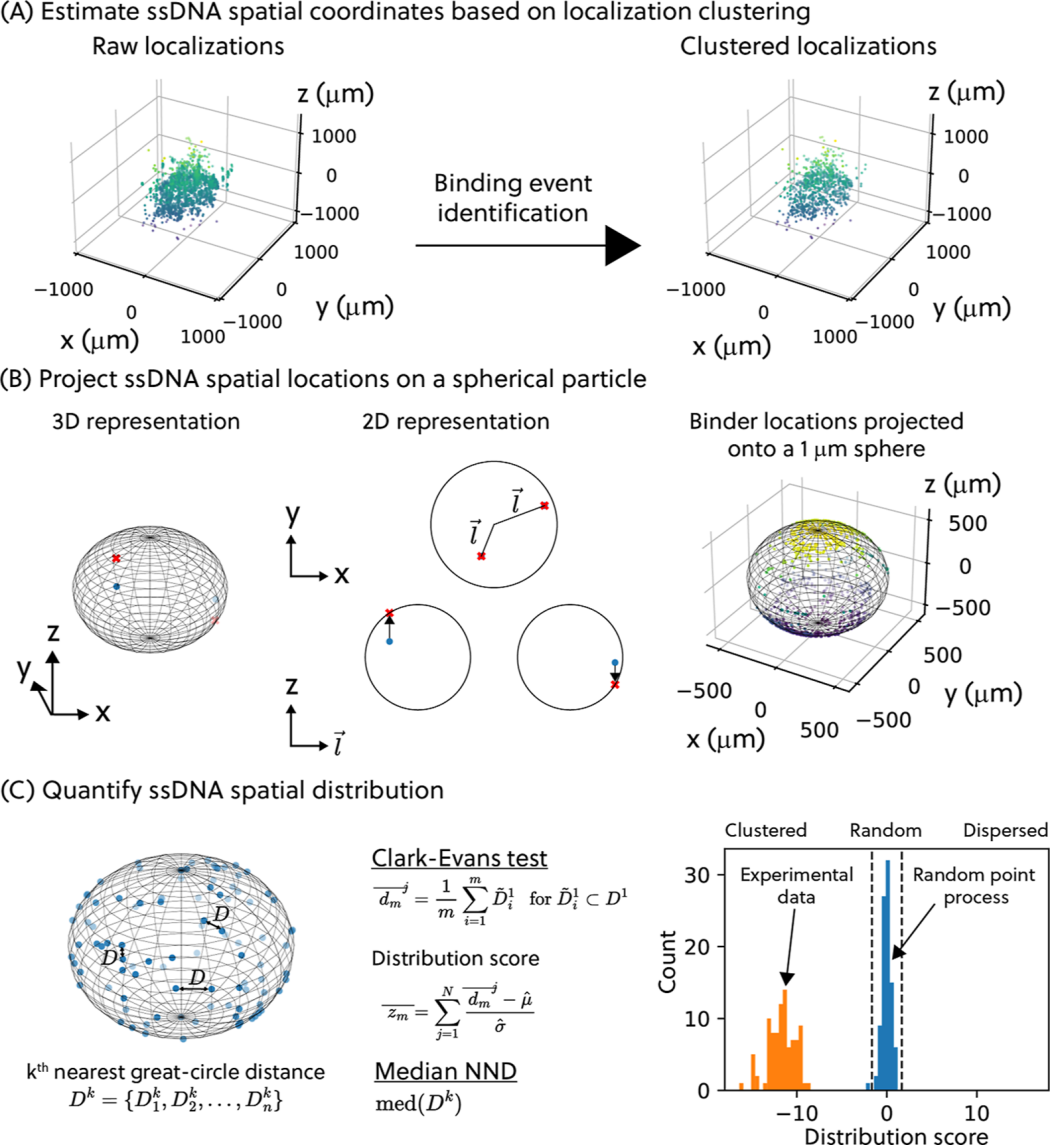
(A) The 3D spatial coordinates of each observed ssDNA molecule were estimated from the localization clustering analysis. (B) To account for the large axial localization uncertainty, the spatial positions of the ssDNA molecules were projected onto the surface of a sphere by adjusting only their axial coordinates. In the example, the blue dot and red cross represent the positions before and after projection, respectively. The rightmost panel shows experimentally obtained ssDNA positions after projection onto the spherical surface. (C) The *k*th order nearest great-circle distance for the ssDNA locations were computed to analyze the spatial distribution of the ssDNA molecules. We computed a distribution score (based on the Clark–Evans test) for each biofunctionalized particle to quantify the spatial distribution of the ssDNA molecules. The distribution scores were compared with those computed from a random point process. Experimental data shown here are particles functionalized with 5 μM complementary ssDNA molecules using streptavidin–biotin coupling.

The projection of ssDNA spatial coordinates on a sphere is performed as follows. The center of the particles is positioned at (0, 0, 0). We first consider a point (exemplified as blue dots in Figure 4B) that has a coordinate of (x, y, z) as a position vector *P⃗* from the origin, as shown in eq 3. Since the lateral localization uncertainty is rather low, only the z-coordinate of the projected point ${\mathrm{P⃗}}^{*}$ (shown as red crosses) should be adjusted such that ${\mathrm{P⃗}}^{*}$ lies on the surface of the sphere, i.e., the magnitude of ${\mathrm{P⃗}}^{*}$ is equal to the radius of the particle *r*. In other words, we weighted the lateral coordinates more heavily and applied correction only to the axial positions.

3 $$\mathrm{P⃗}=\left(\begin{array}{l} x\\ y\\ z \end{array}\right),{\mathrm{P⃗}}^{*}=\left(\begin{array}{l} x\\ y\\ z^{*} \end{array}\right),\mathrm{l⃗}=\left(\begin{array}{l} x\\ y\\ 0 \end{array}\right)$$

A line vector *l⃗* that is in the direction of *P⃗* but lies in the plane *z* = 0 is also defined to aid the illustration of the projection of the points onto a sphere. Using eq 4, we then obtained the projected z coordinates on the surface of the spheres. Note that the sign of the projected z-coordinate is dependent on the sign of the z-coordinate before the projection.

4 $$z^{*}=sgn\left(z\right)\cdot \sqrt{r^{2}-x^{2}-y^{2}}$$

An example of the projected spatial locations of the ssDNA molecules is shown in Figure 4B. Since the assignment of a binding event to the top or bottom half of the sphere depends on its z-coordinate, we considered that the large axial localization uncertainty might introduce errors in this classification and potentially affect the spatial distribution analysis. To investigate this, we performed simulations detailed in Section S2.3. The results show that errors arising from top/bottom misassignments due to axial uncertainty are not a dominant contributor to the overall error in the spatial distribution analysis.

Following that, the kth order nearest neighbor great-circle distances (NND) *D* ^k^ were computed from the spatial coordinates to quantify the spatial distributions of the ssDNA molecules. Great-circle distance is the distance between two points on a sphere measured along the great-circle arc (i.e., the shortest path between the two points on the curved surfaces) between them. Different orders of NND give indications of the length scales at which the ssDNA molecules were distributed. Based on the first-order NND *D* ^1^, we compute a distribution score for each particle based on a statistical test, known as Clark–Evans test,^30^ using eq 5 to eq 7. The Clark–Evans test uses a reference distribution based on the complete spatial randomness (CSR) hypothesis, to which the observed spatial point patterns are compared. Briefly, subsets of NNDs with sample size *m* are chosen and used for analysis to ensure the validity of the test that assumes the independence of the NND. *N* mean NNDs ${\overset{®}{d_{m}\;}}^{j}$ (computed from these subsets) were standardized with the mean μ̂ and standard error σ̂ of NNDs of a CSR point pattern and averaged to give a sample mean NND $\overset{®}{z_{m}\;}$ (denoted as distribution score in this study).

5 $${\overset{®}{d_{m}\;}}^{j}=\frac{1}{m}\sum_{i=1}^{m}{\mathrm{D̃}}_{i}^{1},\text{for}{\mathrm{D̃}}_{i}^{1}\subset D^{1}$$

6 $$\overset{®}{z_{m}\;}=\frac{1}{N}\sum_{j=1}^{N}\frac{{\overset{®}{d_{m}\;}}^{j}-\mathrm{\mu ̂}}{\mathrm{\sigma ̂}}$$

7 $$\mathrm{\mu ̂}=\frac{1}{2\sqrt{\lambda }},\mathrm{\sigma ̂}=\sqrt{\frac{4-\pi }{m4\pi \lambda }},\lambda =\frac{n_{ssDNA}}{A_{part}}$$

In eq 7, λ denotes the density of ssDNA molecules on the particles and can be computed by dividing the DC-quantified number of ssDNA molecules per particle *n*_ssDNA_ with the surface area of the particle *A*_part_. The distribution score is tested against the distribution score of a CSR point pattern at a 5% significance level *z*_0.05_ = 1.65, as shown in eq 8 and illustrated in Figure 4C.

8 $$\begin{array}{llllll} & \overset{®}{z_{m}\;}<-z_{0.05} & & : & & Significant\;clustering\\ & \overset{®}{z_{m}\;}>z_{0.05} & & : & & Significant\;dispersion \end{array}$$

In Section S2.3, we show that errors in the quantified distribution score are dependent on the statistics (DC-quantified number of ssDNA spatial locations), molecular sampling ratio, and localization uncertainties. With the quantified number of ssDNA molecules per particle, the molecular sampling ratio (≈73%; see Figure S12) and the localization uncertainties presented in this article, we expect the distribution score to sufficiently describe the spatial distributions of the conjugated ssDNA molecules. Based on the experimental data and Figure S7, the absolute error in the observed distribution score is estimated to remain below 10 for a true distribution score of −30. However, we emphasize that the observed distribution score also depends on the underlying true spatial organization of the molecules. Its interpretation is therefore not straightforward and requires a detailed understanding of how sampling ratio, localization uncertainties, and true spatial properties interact. As this topic lies beyond the scope of the current work, it is not further addressed here and will be explored in future studies.

The analysis framework outlined in this article was applied to the molecular systems described in the previous section, to study interparticle and intraparticle heterogeneity related to biofunctionalization conditions. Specifically, we investigated the effects of incubation concentration of ssDNA molecules that were conjugated to the particles via either the streptavidin–biotin coupling or the PLL-*g*-PEG-based click-coupling, as shown in Figure 5.

**Figure 5:**
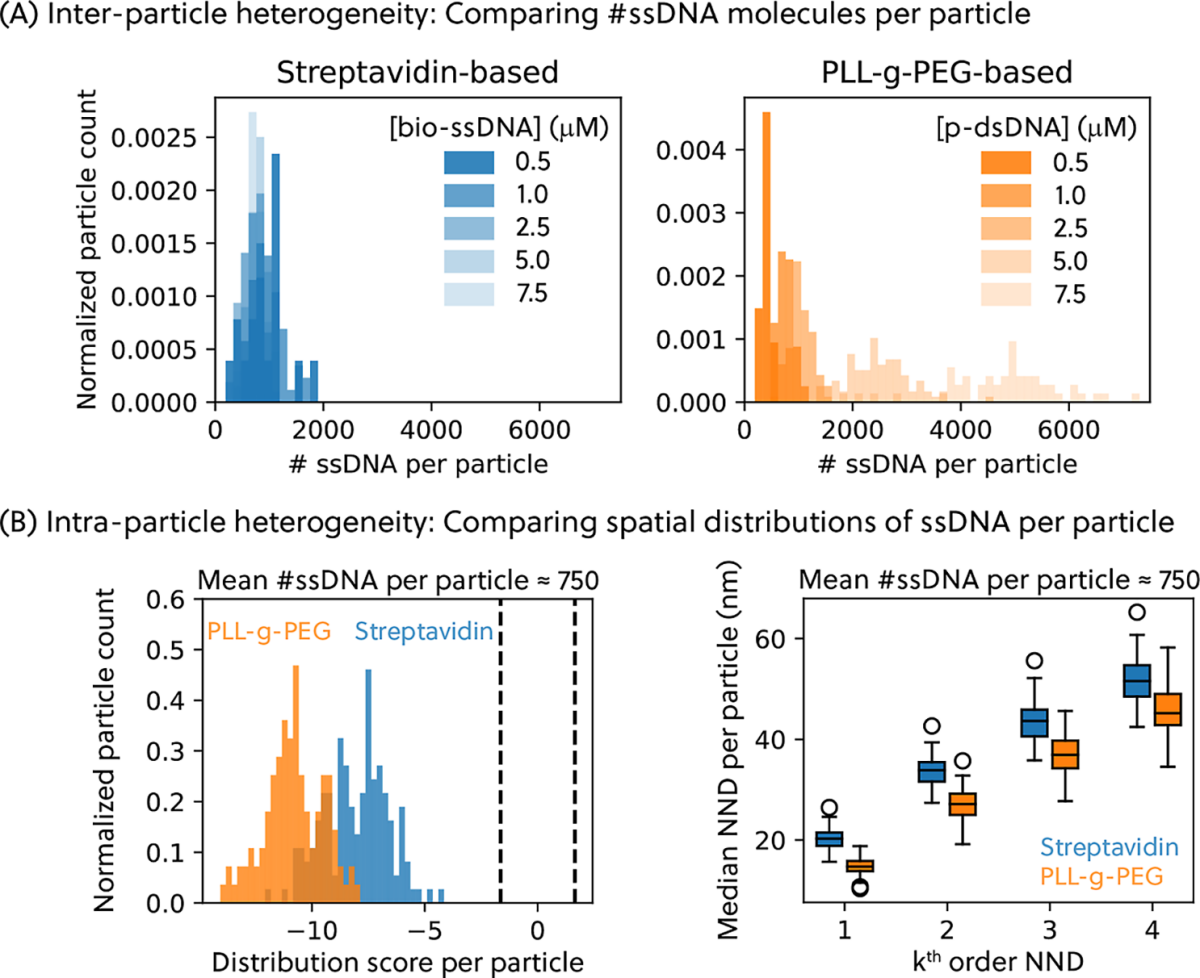
(A) Interparticle spatial heterogeneity was found when comparing the number of ssDNA molecules per particle, obtained from the BiSC approach. (Left) For the streptavidin–biotin coupling, the mean number of ssDNA molecules per particle did not change when the incubation concentration of the biotinylated ssDNA linker (bio-ssDNA) was increased. The ssDNA docking incubation concentration is 100 μM. (Right) For the PLL-*g*-PEG-based click coupling, the average number of ssDNA molecules per particle increased (with wider distributions) with the incubation concentration of the partially double-stranded DNA molecules (p-dsDNA). (B) The spatial distributions of ssDNA molecules on the surface of each particle were analyzed for the intraparticle heterogeneity. For easy comparison, the particles with similar distribution of number of ssDNA molecules per particle were analyzed; see Figure S15. The left panel shows the distribution score $\overset{®}{z_{m}\;}$ per particle, while the right panel shows the median NND per particle. The box represents the 25 to 75% percentile, whiskers 5 to 95%, empty circles the outliers. To aid interpretation, visual representations illustrating how molecular clustering varies across different distribution scores are shown in Figure S17. For all experimental data shown here, the particles were imaged with 500 pM of imager strands.

The data shown here were analyzed from particle ROIs that contain clear particle localization clouds with a round shape corresponding to a spherical particle. Particle ROIs that contain oddly shaped particle localization clouds or that contain no clear particle localization cloud were discarded from the quantification analysis; see Section S2.1. Oddly shaped particle localization clouds can arise from clustered or moving particles. We interpret the particle ROIs that contain no clear particle localization cloud as particles conjugated with a number of ssDNA molecules that were below the quantification limit of this study. The fraction of particle ROIs below the quantification limit is given in Table S4. The quantification limit is dependent on the number of specific localizations on the particle and on the background localizations originating from nonspecific interactions between the imager strands and the glass surface. In the data, different glass surface preparations (see Experimental Section) were needed for the two types of biofunctionalized particles in order to physically adsorb the particles to the surface, resulting in different background localizations. All experiments were performed with the same imager concentration; thus, conditions with higher background resulted in more particles being below the quantification limit.

Figure 5A shows the distributions of the number of ssDNA molecules per particle for different conjugation methods and varying DNA conjugation concentrations. The number of ssDNA molecules represents the number of origami-equivalent, accessible ssDNA docking molecules on the particles. For the streptavidin-coated particles, the number of ssDNA molecules per particle does not increase with increasing biotinylated DNA conjugation concentration, indicating that the quantifiable particles were saturated with ssDNA molecules. This observation is in agreement with the fact that the DNA incubation concentration is above the binding capacity of the streptavidin-coated particles. Surprisingly, the measurements show that the mean number of ssDNA molecules per particle is 3 orders of magnitude lower than the expected number of DNA molecules per particle at saturation binding capacity; see Section S1.1.

We performed several checks to rule out possible artifacts in the ssDNA functionalization procedure of the streptavidin-coated particles. We studied whether particle clustering could play a role in the preparation procedure. However, the number of ssDNA molecules coupled to the particles was the same for particles that were sonicated and particles that were not sonicated prior to ssDNA coupling; see Figure S13. Furthermore, we checked the stability of the binding capacity of the streptavidin-coated particles. We repeated the coupling experiment on freshly bought streptavidin-coated particles, as shown in Section S3.7.2, and found only a small increase in the quantified number of ssDNA molecules per particle. Thus, a loss of binding capacity over time cannot explain the observed 3 orders of magnitude lower number of DNA molecules per particle at saturation.

We hypothesize that molecular accessibility issues may be at the origin of the low localization density observed in the DNA-PAINT experiments on streptavidin-coated particles. The ssDNA docking molecules are coupled to the particles in a two-step procedure; see Figure 1C. In the first step, biotinylated ssDNA linker molecules are coupled to the streptavidin-coated particles, and in the second step, ssDNA docking molecules are hybridized to the linker molecules. In conditions with high densities of ssDNA molecules on the particles (up to 10^5^ molecules per μm^2^), two accessibility issues may appear. First, a high density of ssDNA linker molecules may limit the accessibility for hybridization of ssDNA docking molecules. Second, a high density of ssDNA docking molecules may limit the accessibility for hybridization of ssDNA imager molecules. These hypotheses could be further investigated by studying different functionalization conditions as well as DNA constructs with different lengths. These are topics for further research.

Figure 5A shows the results for the second coupling method: particles biofunctionalized via PLL-*g*-PEG-based click coupling. For this conjugation method, the dibenzocyclooctyne (DBCO)-functionalized ssDNA linker molecules were prehybridized with the ssDNA docking molecules prior to conjugating to the PLL-*g*-PEG-coated particles. A larger mean number of ssDNA molecules per particle with a broader distribution was found with increasing ssDNA incubation concentration. This indicates that more ssDNA molecules were functionalized onto the particles and that a larger interparticle heterogeneity was present in the batch of biofunctionalized particles. The range of number of ssDNA molecules per particle was comparable to the estimated number of ssDNA molecules as calculated in Section S1.2. We postulate that the broad distribution of the number of ssDNA molecules per particle may be caused by the heterogeneous coating of azide-functionalized PLL-*g*-PEG on the particles. Although PLL-*g*-PEG-based systems are easy-to-use, modular platforms for conjugating biomolecules onto surfaces, the attachment of PLL-*g*-PEG molecules on surfaces is dynamic in nature^27^ and mixtures of nonfunctionalized PLL-*g*-PEG molecules and PLL-*g*-PEG molecules with reactive moieties may contribute to spatial molecular heterogeneity.

In terms of intraparticle heterogeneity, we observed a clustered distribution of ssDNA molecules conjugated on particles based on the Clark–Evans test, as shown in Figure 5B. For easy comparison between coupling methods, particles with similar numbers of ssDNA molecules per particle were compared. ssDNA molecules were quantified to be clustered for both conjugation methods, with slightly more clustering observed for the ssDNA molecules conjugated via the PLL-*g*-PEG-based click coupling than those conjugated via the streptavidin–biotin coupling. This is shown as a more negative distribution score for the PLL-*g*-PEG-based system. Similarly, we also found the median first order NND to fourth order NND for the particles prepared via PLL-*g*-PEG-based method to be consistently smaller than those prepared via the streptavidin-based method, further highlighting the more clustered distribution of the ssDNA molecules conjugated via the PLL-*g*-PEG-based approach. To aid interpretation, we included visual representations that illustrate how molecular clustering changes across different distribution scores; see Figure S17.

The observed clustering is in agreement with the fact that the underlying reactive sites are clustered: streptavidin has multiple biotin binding sites and azide-functionalized PLL-*g*-PEG molecules have multiple azide groups per polymer. The clustering of the conjugated ssDNA molecules is further verified by the observation that the clustering degree of the ssDNA molecules increases for increasing numbers of conjugated ssDNA molecules; see Figure S16. However, to grasp why the PLL-*g*-PEG-based system creates a more clustered distribution of ssDNA molecules requires a better understanding of the molecular picture of the system. For a given surface area, the number of ssDNA molecules that can be theoretically functionalized on a streptavidin-coated surface is less than that on a PLL-*g*-PEG-coated surface; see Section S1.3. Therefore, the intermolecular distance of the ssDNA molecules in the PLL-*g*-PEG-based system may be smaller than in the streptavidin-based system, resulting in a more clustered distribution of ssDNA molecules on PLL-*g*-PEG-functionalized particles.

## Conclusions

In this article, we described a single-molecule analysis framework based on 3D DNA-PAINT imaging to quantitatively assess the spatial molecular heterogeneity on biofunctionalized particles. The framework correlates single-molecule data to particles, counts the number of conjugated biomolecules, and quantitatively describes the molecular distribution of conjugated biomolecules. We applied a generalizable method (DBSCAN) to correlate single-molecule data to the particles and implemented a molecular counting methodology (BiSC analysis) that compensates for spatial undersampling or oversampling of biomolecules. Moreover, we applied statistical tests on the observed biomolecular spatial coordinates and quantified the molecular distribution of the conjugated biomolecules. While methods such as DBSCAN, the Clark–Evans test, and other DNA-PAINT-based analyses have previously been used to study biofunctionalized particles, they are typically limited to low-density systems or offer only partial insights into spatial organization. Our approach addressed these limitations by relaxing constraints on imaging parameters and improving access to the spatial localization of coupled biomolecules. Using this framework, we found large differences between particles in terms of the number of conjugated ssDNA molecules, as well as clustering of the conjugated ssDNA molecules, both properties dependent on the bioconjugation method.

The described methodology provides a new strategy for molecular counting and distribution quantification using a single-molecule technique, by employing analysis features that are flexible with respect to experimental parameters (imager concentration and imaging duration) and still provide sufficient statistics for a reliable quantitative assessment of spatial molecular distributions. In the field of DNA-PAINT, qPAINT-based approaches are common for quantifying numbers of biomolecules in a region of interest.^28,31^ However, qPAINT relies on the assumption that no double events occur during the measurement duration, i.e., only single binding events are observed in the region of interest at any time during the measurement. This restricts the experimental conditions and statistics that can be collected over a given time period. Furthermore, a HILO illumination was employed in order to image the whole particle rather than only a small fraction of the particle surface.^13^ The present work implements an imaging method with high event statistics and probing of the full particle surface to allow for optimal assessments of the spatial distributions of conjugated biomolecules. Using the developed methodology, we quantified biomolecule densities up to 2000 μm^–2^. In principle, the simulation-based BiSC analysis is capable of quantifying even higher densities. However, two limitations arise at extremes: at very low densities, the number of binding events becomes too low for robust quantification; at very high densities, molecular accessibility may be hindered due to crowding, limiting the interaction of imager strands with docking sites. These are important topics for future exploration to further extend the applicability and robustness of the methodology.

The described analysis paves the way toward using single-molecule techniques such as DNA-PAINT to evaluate spatial molecular heterogeneity on biofunctionalized particles. The interparticle and intraparticle heterogeneity, which are found to be dependent on the bioconjugation method, are crucial elements to understand and improve the functionality of particle-based applications. For example, studies have shown that spatial heterogeneities on biofunctionalized particles can affect cellular uptake and influence downstream signaling pathways.^32,33^ In regenerative medicine, the spatial patterning of biomolecules on surfaces, mediated by biofunctionalized particles, has been shown to promote osteogenic differentiation of mesenchymal stem cells by modulating focal adhesion formation and mechanotransduction pathways.^34^ A recent study by Vu et al. found a strong correlation between conjugated biomolecular densities and the response behavior of sensing particles.^35^ This highlights the importance of being able to measure heterogeneities of biofunctionalized particles with a single-molecule technique as developed in this paper. In the present work, we propose several hypotheses to explain observed heterogeneities and ways to investigate them experimentally. While orthogonal techniques could provide complementary insights, many commonly available methods either lack the spatial resolution needed to resolve single-molecule features (e.g., ensemble-based techniques like zeta potential) or require substantial instrumentation and experimental optimization (e.g., other SMLM techniques such as stochastic optical reconstruction microscopy (STORM), and photoactivated localization microscopy (PALM)). These directions remain topics for future investigation.

In the future, biofunctionalized particles of varying materials and geometries can be studied, which can be coupled to application-oriented studies that require good control of the biomolecule density and distribution on particles. While the approach is exemplified using 1 μm particles, it remains broadly applicable to particles of varying sizes, provided that information on the localizations can be precisely determined. Furthermore, the described framework is generalizable to biomolecules other than ssDNA molecules by labeling the target biomolecule of interest with a docking ssDNA-functionalized labeling probe, such as protein G, protein M, nanobody, etc.^36–40^ The results in this work also prompt future investigations into the relationship between the molecular density and accessibility of biomolecules using single-molecule analyses. We envision that the 3D DNA-PAINT methodology can become a versatile characterization technique for studying biofunctionalized particles and will help to select and optimize bioconjugation strategies for applications in the fields of targeted drug delivery, particle-based sensors, and regenerative medicine.

## Experimental Section

### Materials and Chemicals

Glass coverslips (22 × 40 mm, thickness no. 1.5, Epredia) were obtained from VWR. Custom-made flow cell stickers with an approximate internal volume of 20 μL were obtained from Grace Biolabs (USA). Dynabeads MyOne Streptavidin C1 and Dynabeads MyOne Carboxylic Acid were purchased from ThermoFisher Scientific, while mPEG-biotin (MW 1 kDa) was purchased from Nanocs. Poly­(l-lysine)-grafted poly­(ethylene glycol) (PLL-*g*-PEG) with a grafting ratio of 3.5 was purchased from SuSoS (Switzerland). The molecular weights of the PLL backbone and PEG side chains are 20 and 2 kDa, respectively. Azide-functionalized PLL-*g*-PEG (PLL-*g*-PEG-N_3_, Nanosoft Biotechnology LLC, USA) is composed of a 15 kDa PLL backbone and 2 kDa PEG chain with a grafting ratio of 5. PBS tablets, NaCl, and Mg_2_Cl were purchased from Sigma-Aldrich, and Tween 20, EDTA, and Tris–HCl were purchased from Merck Life Science. The ssDNA oligonucleotides (standard desalting and HPLC purification for chemically modified DNA) were purchased from IDT (Integrated DNA Technologies). All ssDNA sequences are detailed in Table S6.

PBS buffer was prepared by dissolving 1 tablet of PBS in 200 mL of Milli-Q water. 1 M NaCl dissolved in PBS was used as the high-salt (HS) buffer in this study. The imaging buffer (Buffer B) consists of 10 mM Mg_2_Cl, 5 mM Tris–HCL, 1 mM EDTA, and 0.05% Tween 20.

### Particle Surface Functionalization

#### Streptavidin–Biotin Coupling

2 μL of 10 mg/mL Dynabeads MyOne Streptavidin C1 (1 μm diameter) was incubated with biotinylated ssDNA linker of varying concentrations (between 0.5 μM and 7.5 μM). The mixture was incubated for 45 min at room temperature on a rotating fin. Subsequently, 40 μL of 100 μM 1 kDa mPEG-biotin in PBS was added, and the mixture was incubated for 30 min at RT on the rotating fin. The particle mixture was washed one time with 0.05 vol % Tween-20 in PBS using magnetic separation. After that, 5 μL of 100 μM ssDNA docking strands was added and incubated for 30 min on the rotating fin. The particle mixture was again washed two times and reconstituted in 200 μL of PBS. The ssDNA-functionalized particle solution was sonicated briefly (∼10 s) in a sonic bath before being used. For Figures 1 to 4, the experimental data shown were analyzed from the biofunctionalized particles prepared from 5 μM of biotinylated ssDNA linker.

#### PLL-*g*-PEG-Based Click Coupling

2 μL of 10 mg/mL Dynabeads MyOne Carboxylic Acid (1 μm diameter) was incubated with 1% v/v PLL-*g*-PEG-N_3_/PLL-*g*-PEG mixture solution (final combined polymer concentration of 0.9 mg/mL). The mixture was incubated for 3 h at room temperature on a rotating fin. The particle mixture was washed one time with 0.05 vol % Tween-20 in PBS using magnetic separation. For this conjugation method, the ssDNA docking strands were first prehybridized with the DBCO-functionalized ssDNA linker molecules to prepare a 10 μM stock solution. The prehybridization protocol involves mixing a 4:1 molar ratio of ssDNA docking molecules to DBCO-functionalized ssDNA linker (both at a concentration of 100 μM) in HS buffer on a rotating fin for at least 2.5 h. After that, the washed particle was incubated overnight with varying concentrations of the prehybridized partially double-stranded DNA molecules (between 0.5 μM and 7.5 μM). The particle mixture was again washed two times and reconstituted in 200 μL of PBS. The ssDNA-functionalized particle solution was sonicated briefly (∼10 s) in a sonic bath before being used.

#### Negative Controls

Three types of particles were prepared as negative controls, as described below.

##### Streptavidin–Biotin Coupling

Bare particles were prepared by washing 2 μL of 10 mg/mL Dynabeads MyOne Streptavidin C1 with 0.05 vol % Tween-20 in PBS using magnetic separation. The washed particles were reconstituted in 200 μL of PBS, and sonicated briefly (∼10 s) in a sonic bath before being used. To prepare the PEG-blocked particles, 2 μL of 10 mg/mL Dynabeads MyOne Streptavidin C1 was incubated with 40 μL of 100 μM 1 kDa mPEG-biotin in PBS for 30 min on the rotating fin. After that, the particle mixture was washed, reconstituted in 200 μL of PBS, and sonicated briefly (∼10 s) in a sonic bath before use. Lastly, the particles functionalized with noncomplementary ssDNA molecules were functionalized with the same method as described in the previous section. The ssDNA docking strands described in the method were replaced with ssDNA molecules that have no complementarity to the ssDNA imager strand.

##### PLL-*g*-PEG-Based Click Coupling

Bare particles were prepared by washing 2 μL of 10 mg/mL Dynabeads MyOne Carboxylic Acid with 0.05 vol % Tween-20 in PBS using magnetic separation. The washed particles were reconstituted in 200 μL of PBS and sonicated briefly (∼10 s) in the sonic bath before being used. To prepare the PLL-*g*-PEG-N_3_/PLL-*g*-PEG-coated particles, 2 μL of 10 mg/mL Dynabeads MyOne Carboxylic Acid was incubated with 1% v/v PLL-*g*-PEG-N_3_/PLL-*g*-PEG mixture solution (final combined polymer concentration of 0.9 mg/mL). The mixture was incubated for 3 h on a rotating fin. After that, the particle mixture was washed, reconstituted in 200 μL of PBS, and sonicated briefly (∼10 s) in a sonic bath before use. Lastly, the particles functionalized with noncomplementary ssDNA molecules were prepared with the same method as described in the previous section. The ssDNA docking strands described in the method were replaced with ssDNA molecules that have no complementarity to the ssDNA imager strand.

### Coverslip Functionalization with PLL-*g*-PEG

The glass coverslips were washed by 10 min of sonication in isopropanol and Milli-Q baths, respectively. After drying the coverslips under nitrogen flow, the coverslip surface was oxidized by treating it to 1 min of oxygen plasma, after which a flow cell sticker was attached to the coverslip. 24 μL of 0.5 mg/mL PLL-*g*-PEG in Milli-Q was then immediately added to the flow cell and incubated for approximately 3 h. The solution in the flow cell was then aspirated to remove unbound or loosely bound polymer molecules and replaced with PBS. The functionalized coverslips were used the next day for the streptavidin-coated particles, while the coverslips were used after 1 week for the PLL-*g*-PEG-coated particles.

### DNA-PAINT Imaging and Data Analysis

As a preparation for DNA-PAINT imaging, the functionalized particles were sedimented onto a PLL-*g*-PEG-coated glass coverslip. Before flowing in the particles, the coverslips were flushed with 100 μL of PBS to exchange for fresh buffer. Then, the buffer in the flow chamber was exchanged with the suspension of particles, and the particles were allowed to sediment and attach to the substrate for 15 min. After this procedure, the unattached particles were flushed away with 100 μL of Buffer B, and ATTO647N-labeled imager ssDNA solution (diluted in Buffer B) was added into the flow chamber right before imaging.

3D DNA-PAINT imaging was performed on an Oxford Nanoimager with a HILO configuration. Fluorescence was recorded using a 100x 1.4 NA oil immersion objective. Images were acquired with an exposure time of 100 ms under 67.5m W of 640 nm laser for 30 min. In addition, one frame of image was acquired under 40m W of 488 nm laser before and after the 30-min recordings. The images recorded using a 488 nm laser were used for particle identification. The concentration of the imager was fixed at 500 pM for all experiments. The imager concentration was tuned using the biofunctionalized particle prepared with 5 μM of biotinylated ssDNA linker (streptavidin-based conjugation). By evaluating the quality of the microscopy images (ensuring minimal overlap of PSFs), an imager concentration of 500 pM was found to be optimal and was kept constant for all experiments to allow comparisons between different bioconjugation methods. A representative single-molecule blinking movie is provided in the Supporting Information, with further details described in Section S3.1.

The raw images were then analyzed with ThunderSTORM,^24^ an open-source plug-in in ImageJ, to extract the localizations of the fluorescence emissions. Afterward, the localizations and their properties were used as inputs in a custom-written Python script to analyze the spatial properties (molecular distribution and number of ssDNA molecules) of each biofunctionalized particle. There are typically about 80 particles in the field of view for each data set shown in this work. The *p*-values provided in this work are obtained via Welch’s *t*-test statistic.

## Supplementary Material





## Notes

Supporting information on the molecular systems employed (streptavidin–biotin coupling, PLL-*g*-PEG-based click coupling, and comparison between the bioconjugation methods), details on the developed data analysis (particle localization cloud identification using simulated data and experimental data, BiSC analysis, distribution quantification using Clark–Evans test), additional information and extended data on ssDNA sequences used, calibration of astigmatism lens, control experiments for the PLL-*g*-PEG-based click coupling method, localization uncertainties, molecular sampling ratio in experimental data, effect of presonication and particle stock aging for the streptavidin–biotin coupling, comparison of distribution score for particles with similar distribution of number of ssDNA molecules per particle, relation between number of ssDNA molecules per particle and distribution score, visual representation of molecular clustering (PDF)
Representative video of single-molecule blinking events (AVI)
