# Supplementary material for: Spatial Molecular Heterogeneity on Biofunctionalized Particles Quantified by Three-Dimensional Single-Molecule DNA-PAINT
Source: Langmuir. 2025 Aug 13;41(33):22181–92. doi: 10.1021/acs.langmuir.5c02403 (PMC12392723; doi:10.1021/acs.langmuir.5c02403)
Supplement: Supplementary file 1 [file la5c02403_si_001.pdf]

Supporting Information: Spatial Molecular Heterogeneity on Biofunctionalized Particles Quantified by Three-Dimensional Single-Molecule DNA-PAINT

Wei Shan Tan,[†,‡] Arthur M. de Jong,[¶,‡] and Menno W. J. Prins[*,†,‡,§]

†*Department of Biomedical Engineering, Eindhoven University of Technology, Eindhoven, 5612 AZ, The Netherlands*

‡*Institute for Complex Molecular Systems (ICMS), Eindhoven University of Technology, Eindhoven, 5612 AZ, The Netherlands*

¶*Department of Applied Physics and Science Education, Eindhoven University of Technology, Eindhoven, 5612 AZ, The Netherlands*

§*Helia Biomonitoring, Eindhoven, 5612 AR, The Netherlands*

E-mail: m.w.j.prins@tue.nl

Contents

1 Molecular system **S-4**

1.1 Streptavidin-biotin coupling S-4

1.2 PLL-g-PEG-based click coupling S-4

1.3 Comparison between bioconjugation methods S-6

2 Data analysis **S-7**

2.1 Particle localization cloud identification S-7

2.1.1 Simulated data S-8

2.1.2 Experimental data S-12

2.2 Biomolecule Sampling Compensation (BiSC) analysis S-16

2.3 Distribution quantification: Clark-Evans test S-18

3 Additional information and extended data **S-22**

3.1 Representative single-molecule blinking movie S-22

3.2 ssDNA sequences S-22

3.3 Calibration of astigmatism lens S-23

3.4 Control experiments: PLL-g-PEG-based click-coupling S-24

3.5 Localization uncertainty of the 3D DNA-PAINT localization data S-26

3.6 Molecular sampling ratio in experimental data S-27

3.7 Streptavidin-biotin coupling: Test of hypotheses S-28

3.7.1 Effect of pre-sonication S-28

3.7.2 Effect of aging on the conjugation capability S-29

3.8 Comparison of distribution score for particles with similar distribution of number of ssDNA molecules per particle S-31

3.9 Relation between number of ssDNA molecule per particle and distribution score S-32

3.10 Visual representation of molecular clustering with respect to distribution score S-33

3.11 Simultaneous detection of localizations in a particle ROI S-34

1 Molecular system

1.1 Streptavidin-biotin coupling

Superparamagnetic microparticles coated with streptavidin molecules were functionalized with ssDNA molecules via streptavidin-biotin coupling. The binding capacity of the streptavidin-coated microparticles was calculated based on the information provided by the manufacturer. According to Table S1, the estimated number of conjugated ssDNA molecules or dsDNA molecules per particle is in the order of 10^5 molecules per particle. All ssDNA conjugation concentrations used in this work were above the binding capacity of the streptavidin-coated microparticles, hence we expect full saturation of the microparticles with ssDNA molecules.

Table S1: Binding capacity of the streptavidin-coated microparticles and the expected number of ssDNA or dsDNA molecules per microparticle. * Binding capacity of ssDNA or dsDNA molecules is inversely proportional to the molecular size of the molecule (number of bases).

Properties		
Microparticle concentration	(mg/mL)	10
Microparticle concentration	(beads/mL)	10^{10}
Binding capacity for ssDNA*	(pmol/mg)	500
Binding capacity for dsDNA*	(μg/mg)	20
Microparticle concentration	(beads/mg)	10^9
Binding capacity for dsDNA*	(pmol/mg)	≈ 900
Number of conjugated ssDNA per particle		3×10^5
Number of conjugated dsDNA per particle		5×10^5

1.2 PLL-g-PEG-based click coupling

Magnetic microparticles with negatively-charged carboxylic acid surface groups were used in this study. The particles were first coated with a mixture of PLL-g-PEG and azide-functionalized PLL-g-PEG molecules, followed by the coupling of the partially dsDNA molecules on the low-fouling coating via click chemistry. PLL-g-PEG molecules contain positively-charged lysine backbones and can interact electrostatically with the surface of the microparticle.

Table S2: Geometrical estimation of number of partially dsDNA molecules per microparticle. *Number of conjugated dsDNA molecules is estimated assuming 50% of the azide-functionalized PEG moieties are available to be reacted.

Properties		
Microparticle surface area	(μm^{-2})	3.14
Molar weight of PLL-g-PEG M_{PLL}	(g/mol)	129160
Molar weight of azide-functionalized PLL-g-PEG $M_{\mathrm{PLL,N_3}}$	(g/mol)	56042
Backbone area of PLL-g-PEG A_{PLL}	(nm^2)	124
Backbone area of azide-functionalized PLL-g-PEG $A_{\mathrm{PLL,N_3}}$	(nm^2)	75
Number of azide-functionalized PEG per polymer		21
Total polymer concentration $c_{\mathrm{tot}}^{\mathrm{mass}}$	(mg/mL)	0.9
Mixing ratio (mass concentration) r_{m}		0.01
Mixing ratio (number) r_{n}		0.02
Azide-functionalized PLL-g-PEG density	(μm^{-2})	185
Number of azide-functionalized PLL-g-PEG		5×10^2
Number of conjugated dsDNA per particle*		5×10^3

To estimate the number of PAINT-available ssDNA molecules that could be coupled onto the microparticle, we first compute the density of azide-functionalized PLL-g-PEG molecules on the particle using Eq. S1. The derivation of the equations is described in previous work.[S1] Briefly, the mixing ratio of the azide-functionalized PLL-g-PEG molecule in number r_{n} is calculated from the known properties of the polymer molecules and the experimental parameters. Combined with the estimated area of the PLL backbone, r_{n} is used to calculate the estimated density of azide-functionalized PLL-g-PEG molecules.

$$
\begin{aligned}
r_{\mathrm{n}} &= \frac{r_{\mathrm{m}} c_{\mathrm{tot}}^{\mathrm{mass}}}{(1-r_{\mathrm{m}}) c_{\mathrm{tot}}^{\mathrm{mass}} \frac{M_{\mathrm{PLL,\ N_3}}}{M_{\mathrm{PLL}}} + r_{\mathrm{m}} c_{\mathrm{tot}}^{\mathrm{mass}}} \\
\sigma_{\mathrm{PLL,\ N_3}} &= \frac{r_{\mathrm{n}}}{(1-r_{\mathrm{n}}) A_{\mathrm{PLL}} + r_{\mathrm{n}} A_{\mathrm{PLL,\ N_3}}},
\end{aligned}
\tag{S1}
$$

where r_{m} denotes the polymer mixing ratio in mass concentration, $c_{\mathrm{tot}}^{\mathrm{mass}}$ the total polymer concentration, M_{PLL} and $M_{\mathrm{PLL,N_3}}$ the molar weight of the PLL-g-PEG molecule and azide-functionalized PLL-g-PEG molecule respectively, and A_{PLL} and $A_{\mathrm{PLL,N_3}}$ the PLL backbone area of the PLL-g-PEG molecule and azide-functionalized PLL-g-PEG molecule respectively.

Assuming 50% of the azide-functionalized PEG moieties are available for the click re-

action, we estimate the number of conjugated dsDNA molecules to be in the range of 10^3 molecules per particle, see Table S2.

1.3 Comparison between bioconjugation methods

For both types of ssDNA-functionalized microparticles, the ssDNA molecules are inherently clustered as multiple ssDNA molecules can couple to a single streptavidin or PLL-g-PEG molecule. To compare between the two types of biofunctionalized particles, we consider an area consisting of a single PLL-g-PEG molecule (area of approximately 75nm^2) with 21 azide-functionalized PEG branches, see Fig. S1. Estimating that 50% of the available azide reactive moieties are coupled to a ssDNA, we expect about 10 ssDNA molecules in this given area. On the other hand, roughly 3 streptavidin molecules (with 4 binding pockets) could fit in this area of interest. Assuming at least 1 binding pocket can react with the biotin-labeled ssDNA, we expect about 3 to 9 ssDNA in the given area. This indicates that more ssDNA molecules could be present in the given area of interest for the biofunctionalized microparticles prepared via the PLL-g-PEG-based click chemistry approach. In other words, the intermolecular distances between the ssDNA molecules are shorter in this case, resulting in a more clustered distribution.

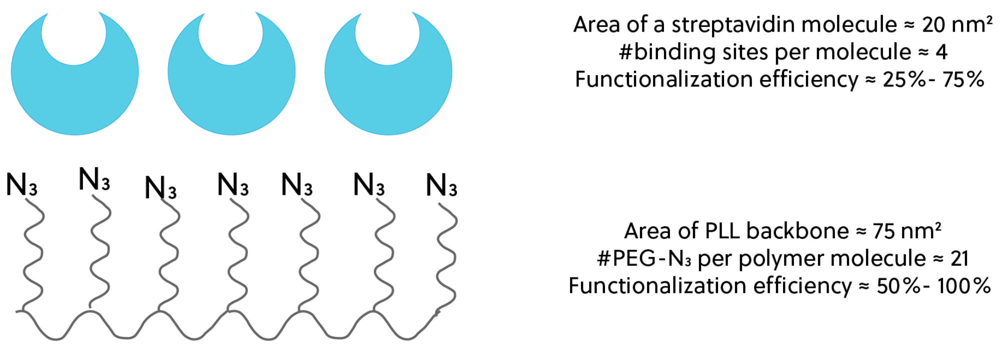

Figure S1: Comparison between streptavidin-coated surface and PLL-g-PEG-coated surface: area of a single molecule, number of coupling sites, and functionalization efficiency.

2 Data analysis

2.1 Particle localization cloud identification

Prior to performing DNA-PAINT imaging on the biofunctionalized microparticles, inherent fluorescence images of the microparticles were first obtained. By running ThunderSTORM analysis on these images, we obtained the estimated center of each microparticle in the field of view. Fig. S2 shows the uncertainties in the localization of the centers for all data shown in this article. After determination of the centers of the microparticles, we drew a 2 x 2 μm^2 region of interest around each microparticle and applied the DBSCAN clustering analysis on these regions to find the localizations related to the microparticle.[S2,S3]

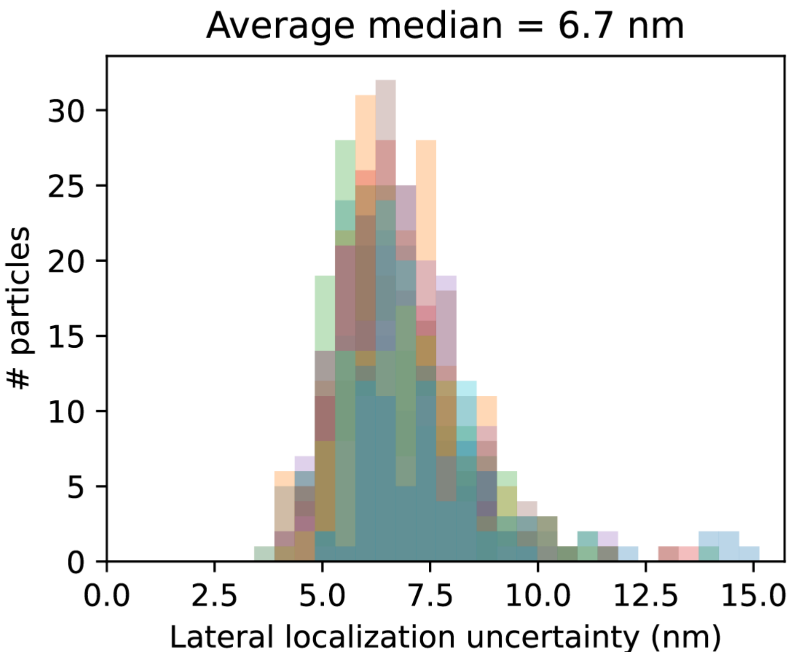

Figure S2: Histograms of the localization uncertainty of the identified particles. The localizations were obtained by running the ThunderSTORM analysis on the inherent fluorescence images of the microparticles. Each color indicates a different dataset.

2.1.1 Simulated data

Localizations related to the microparticle (also referred to as the particle localization cloud) are identified using the DBSCAN clustering analysis. DBSCAN is a density-based clustering algorithm, i.e., the algorithm groups together points that are spatially close together (given the search radius ϵ and the minimum number of points in the neighborhood minPts). Errors from this analysis step would lead to errors in the quantification of the number and molecular distribution of the ssDNA molecules on the microparticles.

To study the possible sources of error in the particle localization cloud identification step, we simulated the localizations in a 2 x 2 μm^2 region of interest containing one microparticle in the center, or a 4 x 2 μm^2 region of interest containing two microparticles. The number of localizations per microparticle are not simulated based on hybridization kinetics between the imager ssDNA and docking ssDNA; instead, we set the number of localizations per microparticle to a given value (referred to as n_{true}).

In both regions of interest, we included noise in the simulated data based on a given signal-to-noise ratio. The signal-to-noise ratio is given by the ratio between the number of specific localizations and localizations originating from noise. Following the generation of localizations, DBSCAN clustering is performed on the localizations to obtain particle-related localizations n_{DBSCAN}. The relative error E in particle localization cloud identification is defined in Eq. S2.

$$\text{E} = \frac{|n_{\text{DBSCAN}} - n_{\text{true}}|}{n_{\text{true}}} \tag{S2}$$

Table S3: Input parameters that were kept constant in the simulation.

Simulation	Variable	n_{true}	ϵ	minPts
1 particle	Signal-to-noise & n_{true}	-	0.1	50
1 particle	Signal-to-noise & ϵ	$1e3$	-	50
1 particle	Signal-to-noise & minPts	$1e3$	0.1	-
2 particle	d & signal-to-noise	$1e3$	0.05	50

In Fig. S3(A)(i), we investigated the effect of signal-to-noise ratio, n_{true} and DBSCAN

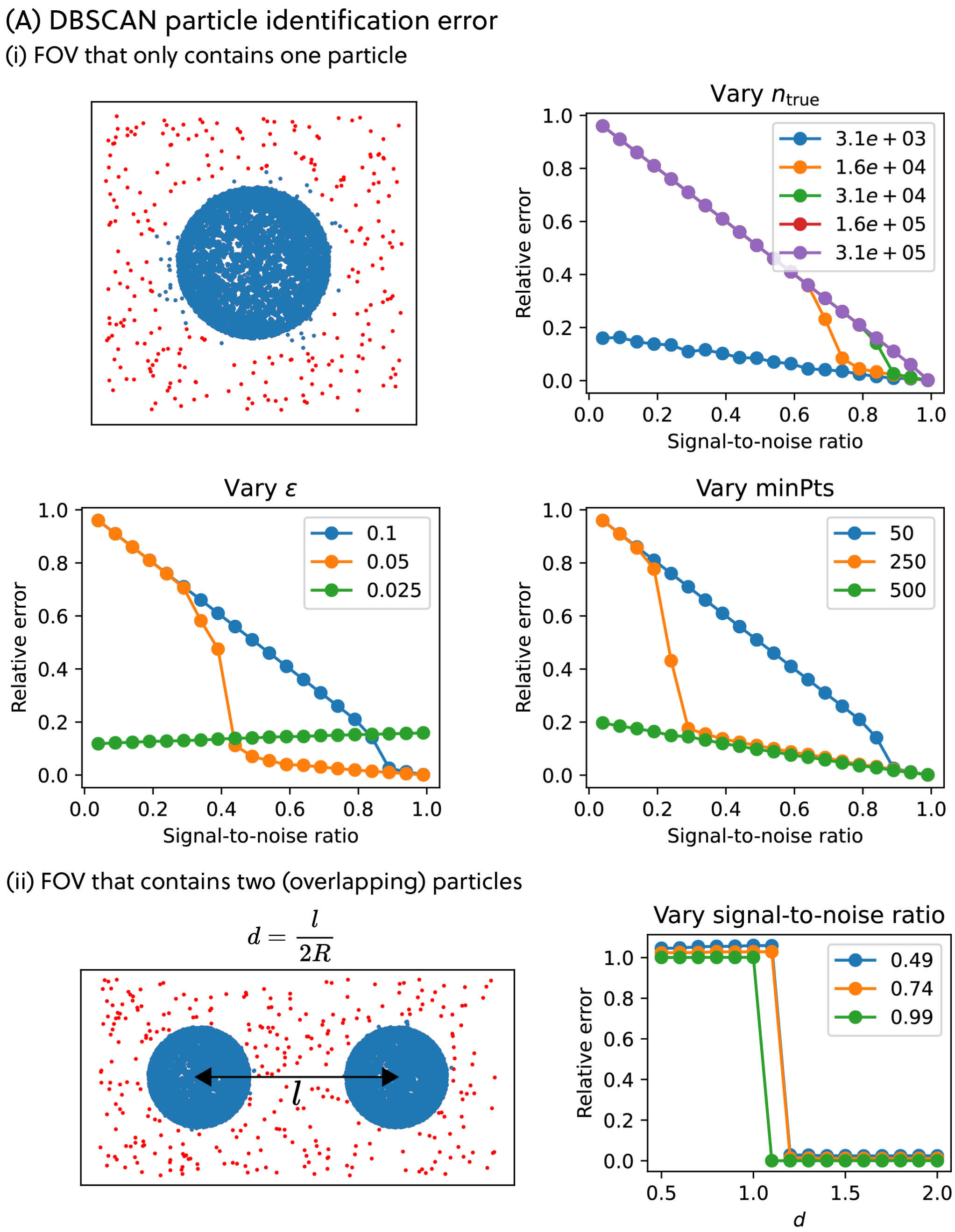

Figure S3: Possible sources of error in particle identification step using DBSCAN investigated using simulated data. (i) Simulated localization data with only one particle in the FOV (blue dots are specific localizations while red dots are noise). The relative error in DBSCAN particle identification decreases when signal-to-noise ratio increases. The relative error is also impacted by the number of localizations per particle n_{true}, and the DBSCAN parameters employed ϵ and minPts. (ii) Simulated localization data with two particles in the FOV (blue dots are specific localizations while red dots are noise). There is a center-to-center distance l threshold at which the DBSCAN cannot distinguish between the localization data of the two particles. The signal-to-noise ratio has minimal impact on the relative error in this case. The input parameters that were kept constant for the results shown are given in Table S3.

clustering parameters (ϵ and minPts) on the relative error of particle localization cloud identification. We found that lower signal-to-noise ratio generally gives rise to higher relative error. However, the extent of the signal-to-noise ratio dependence on the relative error is greatly influenced by the true number of specific locations n_{true} and the DBSCAN parameters employed. For higher n_{true}, the quality of DBSCAN clustering deteriorates rapidly with decreasing signal-to-noise ratio, giving larger relative error in particle localization cloud identification. To interpret the observation, we have to realize two points:

1. Signal-to-noise ratio is defined as the ratio between specific localizations on the microparticle and localizations originating from background or noise. This means that, for the same signal-to-noise ratio, higher n_{true} also results in more noise localizations.

2. Since DBSCAN is a density-based clustering algorithm, it may be easier to induce errors when more localizations (both specific and noise) are found in the region of interest.

This indicates that, for microparticles functionalized with a large number of ssDNA molecules, the quality of the particle cloud identification is extremely sensitive to noise. Therefore, it is utmost important to ensure that background localizations are well suppressed during DNA-PAINT imaging.

Furthermore, it is logical that the DBSCAN parameters affect the quality of the particle cloud identification. As with n_{true}, the relative error of particle cloud identification generally decreases when signal-to-noise increases when different ϵ or minPts values are used as inputs for DBSCAN with one exception. However, when very low ϵ is used, the relative error slightly increases with increasing signal-to-noise ratio. In other words, when a search radius much smaller than the actual shape of the localization cloud is used, the error in localization cloud identification would remain high regardless of the signal-to-noise ratio of the data. Another thing to note in Fig. S3(A)(i) is that the relative error in particle cloud identification can significantly decrease with a slight increase in signal-to-noise ratio (a kink could be observed

in the relative error against signal-to-noise ratio) depending on the true number of PAINT localizations per microparticle n_{true} and the DBSCAN clustering parameters ϵ and minPts. This highlights the importance of choosing suitable DBSCAN parameters for a given dataset to ensure the actual particle localization cloud is being selected.

In Fig. S3(A)(ii), we investigated the effect of two possibly overlapping particles on the quality of the particle cloud identification. In experiments, it is often not possible to ensure that the microparticles are not in close proximity with each other. To understand the distance threshold between two particles at which the two particles can no longer be distinguished from each other, we simulated two microparticles (with the same radius R and n_{true}) in a 4 x 2 μm region of interest and defined a normalized distance parameter d between the particle as given in Eq. S3.

$$d = \frac{l}{2R} \tag{S3}$$

where l denotes the center-to-center distance between the two particles. For the same n_{true}, and DBSCAN clustering parameters used, the n_{DBSCAN} for both particles are the same until the algorithm can no longer differentiate between the PAINT localizations between the two particles. Thus, the relative error in particle cloud identification shown in Fig. S3(A)(ii) are the relative identification errors for one particle. The simulation results showed that there is a threshold for the distance at which the clustering algorithm can no longer distinguish between the specific localizations belonging to the two particles. The threshold is slightly larger than 1 ($d_{\text{threshold}} = 1.2$, corresponding to center-to-center distance l of 2.4 μm), and can only be minimally improved with very high signal-to-noise ratio. Therefore, it is only possible to identify particle localization clouds accurately when there is no overlap between neighboring particle localization clouds.

Overall, to select the particle localization cloud with good precision, it is important to tune the DBSCAN clustering parameters ϵ and minPts depending on the quality of the 3D DNA-PAINT imaging data obtained. However, for good comparison between different datasets, the same DBSCAN clustering parameters are typically used to analyze micropar-

ticles prepared with different bioconjugation method and conditions. In other words, the DBSCAN parameters may not be optimal for some datasets. To prevent incurring errors in downstream analysis, each particle region of interest and the found particle localization cloud in all experimental datasets was carefully inspected in this study.

2.1.2 Experimental data

By carefully inspecting the regions of interest (ROIs) containing microparticles in the experimental datasets, we found a portion of particle ROIs that either contain no clear particle localization cloud or contain overlapped or weirdly-shaped particle localization clouds. These particle ROIs were discarded from downstream analysis (molecular quantification). Interestingly, the fraction of discarded particle ROIs decreased with increasing ssDNA incubation concentration for the streptavidin-biotin coupling method, while the discarded particle fraction did not change with the ssDNA incubation concentration for the PLL-g-PEG-based click coupling method, as shown in Fig. S4.

To study the possible origin of the difference between the two types of biofunctionalized microparticles, we compared the total number of localizations in the discarded particle ROIs to the total number of localizations in the background ROIs (2 x 2 μm ROIs that do not contain a microparticle), see Fig. S5(A). For the microparticles prepared with PLL-g-PEG-based click coupling, the total number of localizations in the discarded particle ROIs increases with increasing ssDNA incubation concentration. On the other hand, for the microparticles prepared via the streptavidin-biotin coupling, not only is there no dependency of the total number of localizations on the ssDNA incubation concentration, the discarded particle ROIs have total number of localizations similar to that of the background ROIs. These discarded particle ROIs contain no clear particle localization cloud or may have particle localization clouds that were not distinguishable from the background.

By setting a threshold on the total number of localizations per ROI using the background ROIs of each dataset (median + std), we classified the discarded particle ROIs into two

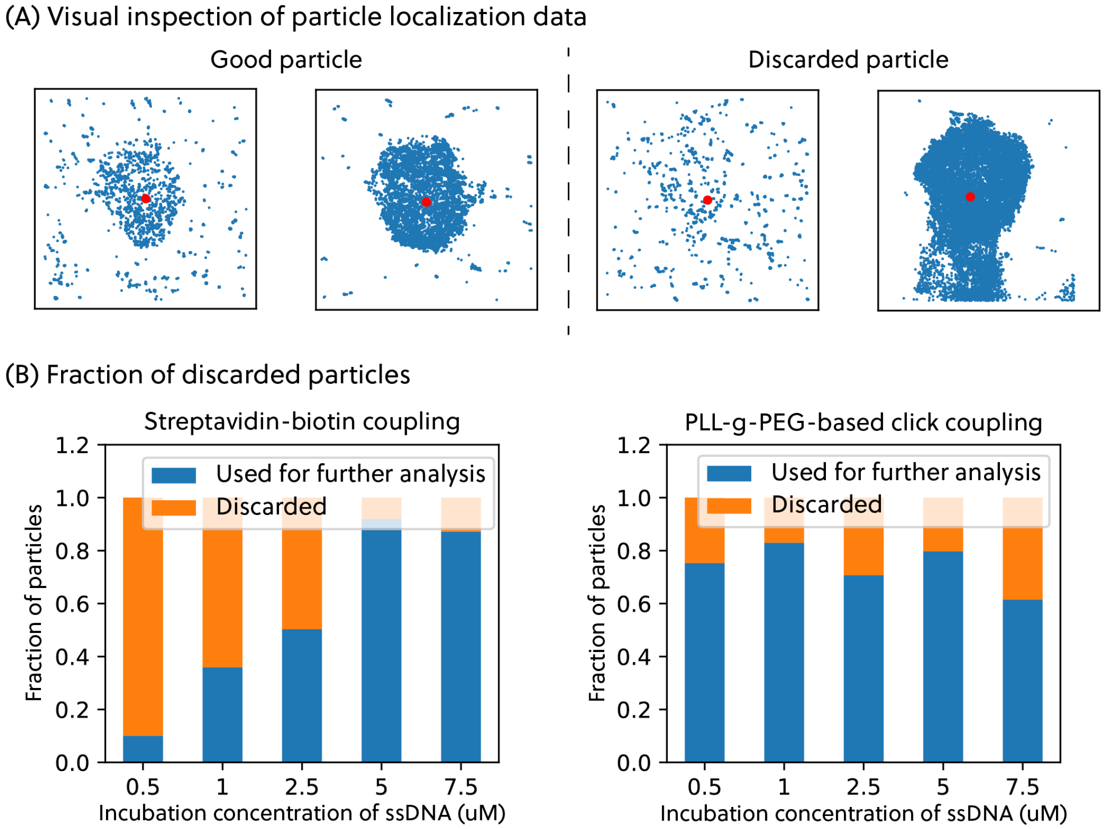

Figure S4: (A) All particle ROIs are carefully inspected to identify reliable particle localization data. Only particle localization clouds that are approximately spherical are considered for further analysis. (B) The fraction of particles that were discarded decreases for increasing ssDNA incubation concentration for the streptavidin-based conjugation, while the discarded particle fraction remains somewhat the same for the PLL-g-PEG-based conjugation.

groups, as shown in Fig. S5(B). The discarded particle ROIs above the threshold contain particle cloud localizations that seem "deformed" (not round as expected from a spherical particle). We hypothesize that the "deformed" particle localization clouds originate from microparticles that were moving during the imaging or microparticles that were clustered. It is not possible to precisely identify the particle localization cloud using DBSCAN. On the other hand, the discarded particle ROIs below the threshold that contain no clear particle localization cloud were interpreted as microparticles conjugated with a number of ssDNA molecules that are below the quantification limit. The fraction of particle ROIs that are below the quantification limit for the data shown in Fig. 5 is shown in Table S4. The imager concentration could be increased to obtain a clearer particle localization cloud in the ROI.

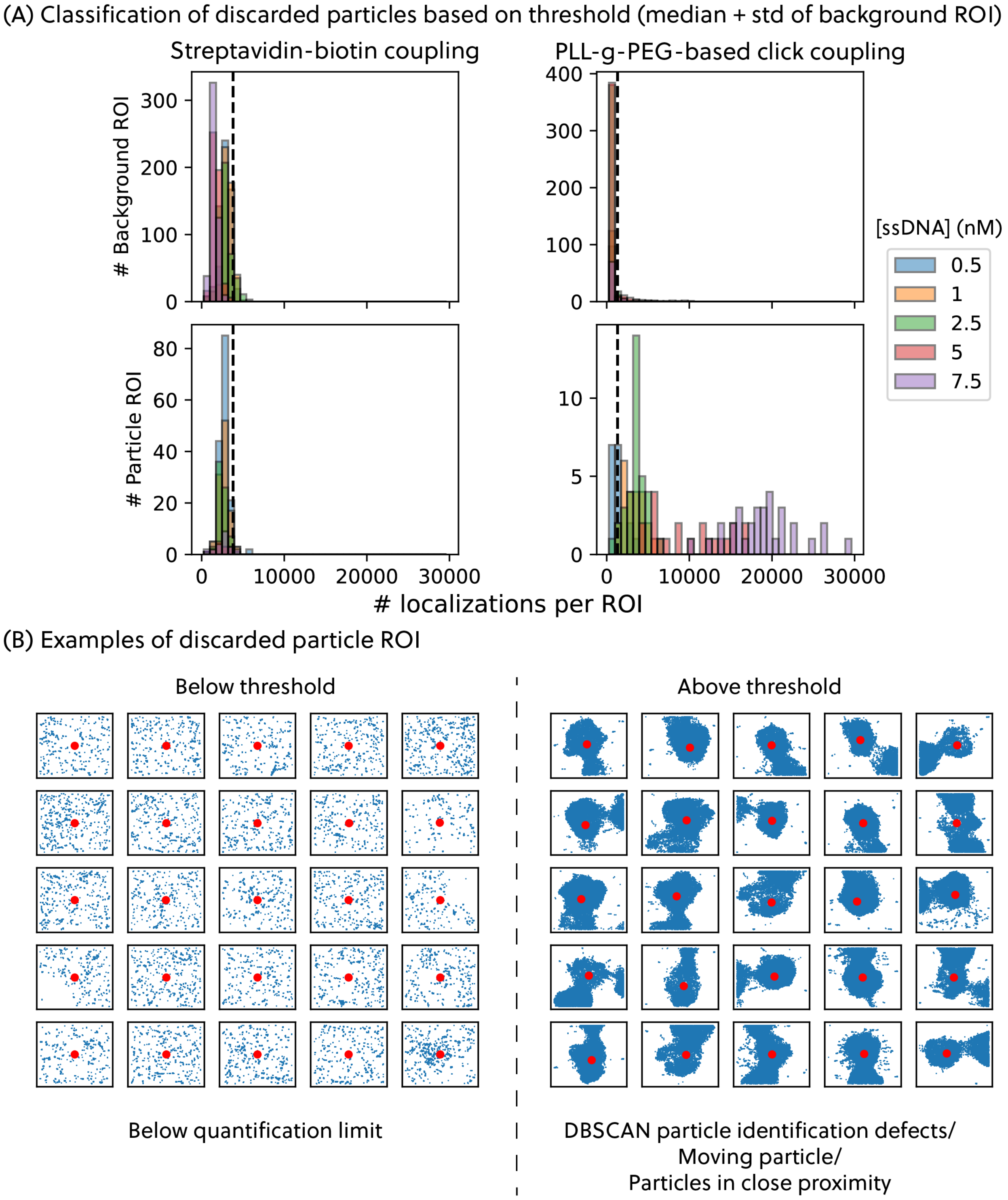

Figure S5: (A) The discarded particles were further distinguished by classifying them according to background ROIs (median + std). (B) Examples of the discarded particle ROIs are shown. Particle ROIs that are below the background threshold are considered as microparticles functionalized with a number of ssDNA molecules below the quantification limit. Particle ROIs that are above the background threshold are considered as DBSCAN particle identification defects, a moving particle, or particles that were in close proximity.

This is not done, however, to ensure consistency and comparability with the other datasets obtained in this study.

Table S4: Fraction of particle ROIs below quantification limit for the data shown in Fig. 5.

Coupling method	ssDNA incubation concentration (μM)	# imaged particles	Fraction of particle ROI below quantification limit	Fraction of particle with irregular shapes (above threshold)
Streptavidin-biotin	0.5	179	0.85	0.04
Streptavidin-biotin	1	170	0.62	0.02
Streptavidin-biotin	2.5	149	0.46	0.03
Streptavidin-biotin	5	162	0.03	0.05
Streptavidin-biotin	7.5	171	0.02	0.11
PLL-g-PEG-based	0.5	69	0.03	0.22
PLL-g-PEG-based	1	135	0	0.17
PLL-g-PEG-based	2.5	116	0	0.29
PLL-g-PEG-based	5	104	0	0.20
PLL-g-PEG-based	7.5	83	0	0.39

2.2 Biomolecule Sampling Compensation (BiSC) analysis

Biomolecule Sampling Compensation (BiSC) analysis was first developed in the previous work, named as Compensation for Binder Undersampling.[S1] The previous analysis methodology was developed to compensate for the undersampling of biomolecules of a high-density surfaces. In this article, depending on the imager concentration used, the ssDNA molecules on each microparticle may be undersampled (not all ssDNA molecules were probed) or oversampled (a single ssDNA molecule was probed multiple times), see Fig. S6. Since the BiSC method relies on the relationship between the number of binding events per biomolecule and the true molecular density, it can equally capture oversampling effects. An increase in binding events due to molecular oversampling is accounted for within the same analysis framework. Thus, the analysis methodology was renamed as Biomolecule Sampling Compensation (BiSC) in this article.

The analysis is based on Monte Carlo simulations that simulate the hybridization events between the imager strand and docking strands (the ssDNA molecules of interest). In the simulation, signal time traces of each ssDNA molecule per microparticle were generated with 100 ms integration time. Then, the number of binding events observed in each time trace is summed to obtain the total number of observed events for a given microparticle. By varying the number of ssDNA molecules per microparticle, a linear relationship between the number of ssDNA molecules per microparticle and the total number of observed binding events was found and used to inversely estimate the number of ssDNA molecules per microparticle in experimental data. The simulation parameters are summarized in Table S5.

This method is preferred over the more established quantitative PAINT (qPAINT) approach because qPAINT assumes that no simultaneous binding events occur within the region of interest, which typically requires lower imager concentrations and longer measurement times to satisfy for high-density systems. In contrast, the BiSC method relaxes this assumption by allowing for the possibility of multiple binding events in a single frame. A natural question arises: how can multiple binding events within a small region of interest be

reliably distinguished in a single frame? The answer depends on the diffraction limit and the physical size of the region. The diffraction limit sets a minimum distance between emitters such that their point spread functions (PSFs) can be resolved as separate localizations. In our previous work,[S1] we estimated that the maximum emitter density per frame that avoids significant PSF overlap is approximately 3.0 μm^{-2}. Given that the region of interest in this study corresponds to the particle size (about 1 μm), up to three emitters can be reliably distinguished per frame.

Table S5: Simulation parameters for BiSC analysis.

Simulation parameter		Value
Molecular dissociation rate k_{off}	(s^{-1})	1
Molecular association rate k_{on}	($M^{-1}s^{-1}$)	10^6
Imager concentration c_{img}	(M)	5×10^{-7}
Simulation duration	(min)	30
Integration time	(s)	0.1
Number of ssDNA molecules per particle	(-)	10 to 3000

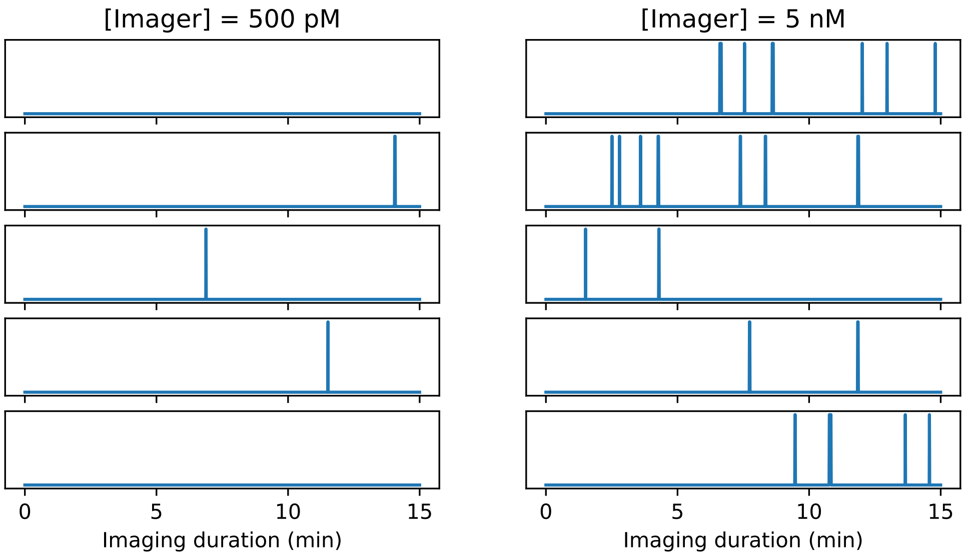

Figure S6: Simulated binding events over a 15-minute period for individual ssDNA docking molecules at varying imager concentrations show distinct sampling regimes. At 500 pM, each docking strand is either probed once or not at all, leading to molecular undersampling. In contrast, a tenfold increase in imager concentration results in each docking strand being probed at least once, leading to molecular oversampling.

2.3 Distribution quantification: Clark-Evans test

To quantify the spatial distribution of the ssDNA molecules on the microparticles, we first obtained the spatial locations of each ssDNA molecule by averaging the localizations belonging to a single binding event. Then, the spatial locations of each ssDNA molecule were adjusted such that they lie on the surface of a sphere (given the radius of the microparticle). In other words, the ssDNA molecules were projected onto the surface of a sphere. The projection is required due to the larger axial localization uncertainties. Afterwards, Clark-Evans test, a nearest-neighbor distance-based statistical test, was performed on the ssDNA spatial locations.[S4] The test compares the average nearest-neighbor distances of the spatial locations to the expected nearest neighbor distance and determines whether the observed spatial point pattern is randomly distributed, significantly clustered, or significantly dispersed based on the quantified distribution score. Note that the distances between the ssDNA molecules were computed as the great-circle distances, i.e. the distances between two points on a sphere measured along the great-circle arc.

In previous work, we found that the molecular sampling ratio greatly impacts the molecular distribution quantification.[S1] To verify this observation, we simulated spatial locations (x, y, and z) of ssDNA molecules on a microparticle given a certain molecular distribution score (random, clustered or dispersed). For a given molecular sampling ratio (fraction of ssDNA molecules probed), localizations per probed ssDNA spatial location were generated. The number of localizations per ssDNA molecule corresponds to the binding time, i.e., how long an imager strand interacts and binds to a ssDNA molecule. The binding time t_b was sampled from an exponential distribution with mean bound state lifetime of 1 s, thus the number of localizations per probed ssDNA molecule n_loc is given by Eq. S4.

$$n_\mathrm{loc} = \frac{t_\mathrm{b}}{t_\mathrm{exposure}} \tag{S4}$$

where t_exposure denotes the exposure time used in the simulation (100 ms). The simulated

localizations per microparticle were then analyzed with the same analysis workflow as the experimental data. Absolute error in molecular distribution quantification is defined as the absolute difference between the observed distribution score and the true distribution score (as computed from the true spatial locations of the ssDNA molecules). Here, the true distribution scores for the three cases of clustered distribution, random distribution and dispersed distribution are -30, 0.02 and 35 respectively.

In Fig. S8(A), we found that the molecular sampling ratio indeed impacts the error in quantifying molecular distribution. As per previous work, a decrease in sampling ratio causes a shift in the observed distribution towards zero, i.e. the observed ssDNA molecules appear to be randomly distributed. Therefore, the sampling ratio only impacts the case where the true molecular distribution of the ssDNA molecules is not random. However, we found that the molecular sampling ratio affects the quantification error more when there is more ssDNA molecules per microparticle, which contradicts previous finding. It is good to note a key difference between results shown from previous work and those shown here. Previous work focused on studying the effect of molecular sampling ratio, hence the sampled spatial locations were directly quantified for their distribution (without generating the PAINT localizations). Here, PAINT localizations were generated from the sampled spatial locations, after which the Clark-Evans test was used to quantify the molecular distribution of the observed molecules.

For cases where there is a large number of ssDNA molecules per microparticle, the absolute error in distribution quantification is large despite having high molecular sampling ratio. We attribute this finding to the high spatial density of molecules of which their spatial locations cannot be precisely determined due to the large localization uncertainties (relative to the distances between the ssDNA molecules). The large quantification error is therefore a consequence of localization uncertainties at high spatial molecular density. This is supported by the simulation results in Fig. S8(B). On the other hand, the axial localization uncertainty has minimal effect on the quantification error; the increase in quantification error is purely

a consequence of the increase in number of ssDNA molecules per microparticle. Since the projection of the probed ssDNA molecules onto a sphere relies on the estimated xy-positions of the probed ssDNA molecules, we expect that the observed distribution score to deviate more from the true distribution score when the microparticle was functionalized with a large number of ssDNA molecules.

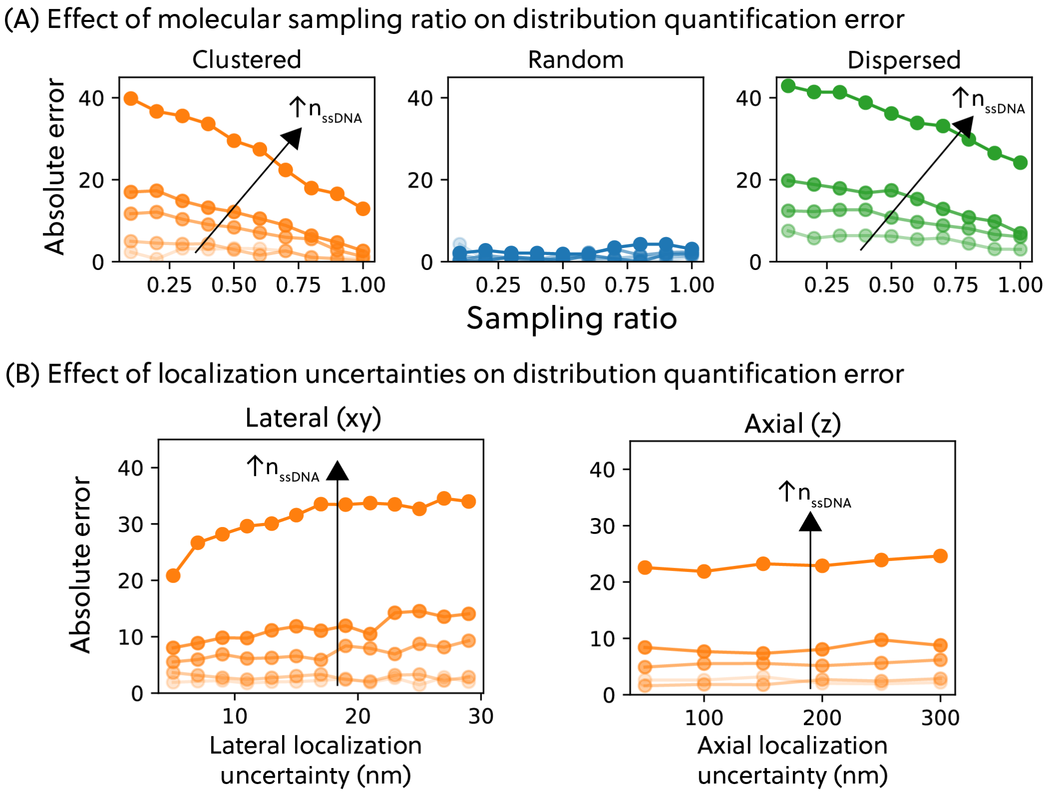

Figure S7: Effect of (A) molecular sampling ratio and (B) localization uncertainties on the error in quantifying molecular distribution. Here, biomolecular spatial locations are simulated and sampled. Localizations are generated around the sampled molecular positions based on specified lateral and axial localization uncertainties. Localization clustering, spherical projection, and spatial distribution analysis are then applied to the generated data. The absolute error represents the difference between the observed and true distribution scores. In (A), the localization uncertainties are set as 5 nm (lateral) and 50 nm (axial). In (B), only the case where the ssDNA molecules were clustered is simulated and the molecular sampling ratio is kept constant as 0.7. In both simulations, the number of ssDNA molecules per microparticle is set as [50, 100, 500, 1000, 5000].

For the experimental data shown in this article, the molecular sampling ratio is approximately 73%, and the lateral and axial localization uncertainties are 6 nm and 84 nm

respectively, see Fig. S11 & S12. Therefore, we do not expect a large error in the quantification of molecular distribution, and the quantified distribution score could still sufficiently describe the spatial distributions of the conjugated ssDNA molecules.

3 Additional information and extended data

3.1 Representative single-molecule blinking movie

A representative 60-second snippet of a single-molecule blinking movie is provided as Supplementary Information. The video shows streptavidin-coated microparticles functionalized with biotinylated ssDNA linker at a concentration of 7.5 µM. Details on the sample preparation and DNA-PAINT imaging conditions are provided in the Experimental Section of the article.

3.2 ssDNA sequences

Table S6 shows the ssDNA molecules that were used in this work. For the streptavidin-biotin coupling, Sequence #1 is first coupled to the microparticle. Thereafter, Sequence #3 or Sequence #4 are hybridized to Sequence #1 to prepare the complementary ssDNA-conjugated microparticles or the non-complementary control microparticles respectively. For the PLL-g-PEG-based click coupling, Sequence #2 is first pre-hybridized with Sequence #3 or Sequence #4 to prepare the complementary ssDNA-conjugated microparticles and the non-complementary control microparticles respectively. Sequence #5 is the ssDNA imager strand.

Table S6: ssDNA sequences used in this work.

#	5' end group	Sequence	3' end group
1	None	`TAG TCA GGT TGG ATG TCT AC`	Biotin
2	None	`TAG TCA GGT TGG ATG TCT AC`	DBCO
3	None	`GTA GAC ATC CAA CCT GAC TAC GTG ACA TTA TTA CAC`	None
4	None	`GTA GAC ATC CAA CCT GAC TAC GTG AGT AAT AAT GTT`	None
5	ATTO647N	`TTG TAA TAA TG`	None

3.3 Calibration of astigmatism lens

To analyze the 3D DNA-PAINT imaging data, the astigmatic lens must be calibrated to accurately correlates the shape of the deformed PSF to its height. The calibration of the lens is performed by imaging fluorescent beads (diameter 20 nm) in Buffer B at a laser wavelength of 640 nm. The z-position of the stage was varied with an interval of 10 nm and fluorescence images of the beads were captured. By running the 3D calibration analysis on the acquired images using ThunderSTORM, the parameters used for the polynomial fit of emitters were obtained, as shown in Fig. S8. The calibration file was used to analyze all 3D DNA-PAINT data shown in this work.

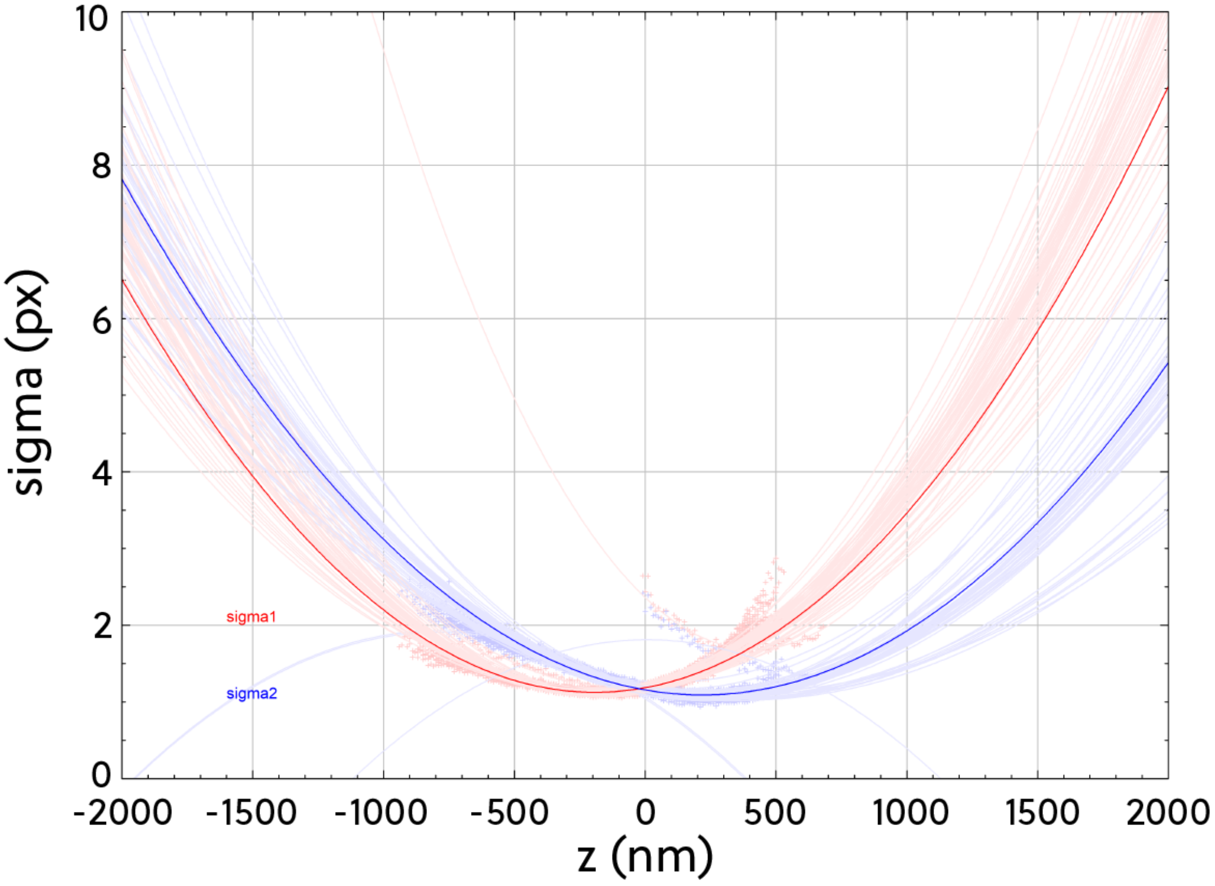

Figure S8: Calibration of the astigmatic lens using fluorescent beads. From the data of PSF width (x and y - sigma1 and sigma2) against z-height, the parameters that were used for the polynomial fit of emitters were obtained.

3.4 Control experiments: PLL-g-PEG-based click-coupling

To confirm successful coating of PLL-g-PEG on the carboxylated particles, we performed zeta potential measurements in phosphate-buffered saline. After coating, the zeta potential of the particles shifted toward zero, indicating a reduction in surface charge, see Fig. S9. This shift is attributed to the neutralization of the negatively charged carboxylic groups by the neutrally charged PLL-g-PEG molecules, confirming effective coating of the particles.

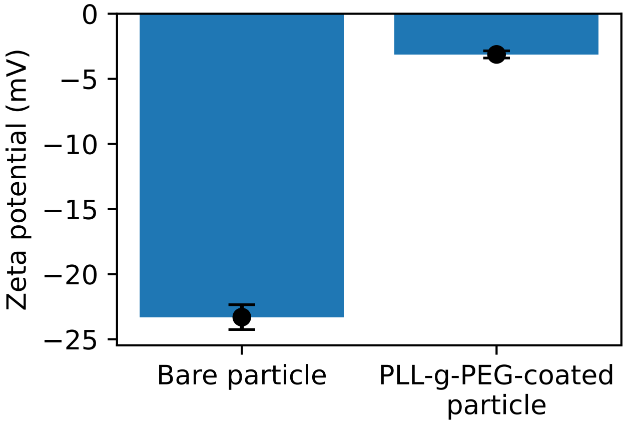

Figure S9: Zeta potential measurements were performed on bare particles with negatively charged surface carboxylic groups and on PLL-g-PEG-coated particles. Each condition was measured in triplicate, and the error bars represent the mean and standard deviation. The results show that the PLL-g-PEG coating effectively neutralizes the surface charge of the particles.

In Fig. S10, we compared the number of localizations in particle ROI for microparticles prepared in different conditions: (1) Bare microparticles with surface carboxylic acid groups, (2) microparticles coated with a mixture of low fouling polymer PLL-g-PEG and azide-functionalized PLL-g-PEG, (3) microparticles coated with PLL-g-PEG polymer and functionalized with non-complementary ssDNA docking strands, and (4) microparticles coated with PLL-g-PEG polymer and functionalized with complementary ssDNA docking strands. Condition 1 to 3 serve as negative controls. By analyzing these microparticles, we found significant differences (p-value less than 0.001) between Condition 4 and the other conditions, thus giving confidence that the 3D DNA-PAINT localization data obtained in the microparticles functionalized with complementary ssDNA docking strands are specific interactions.

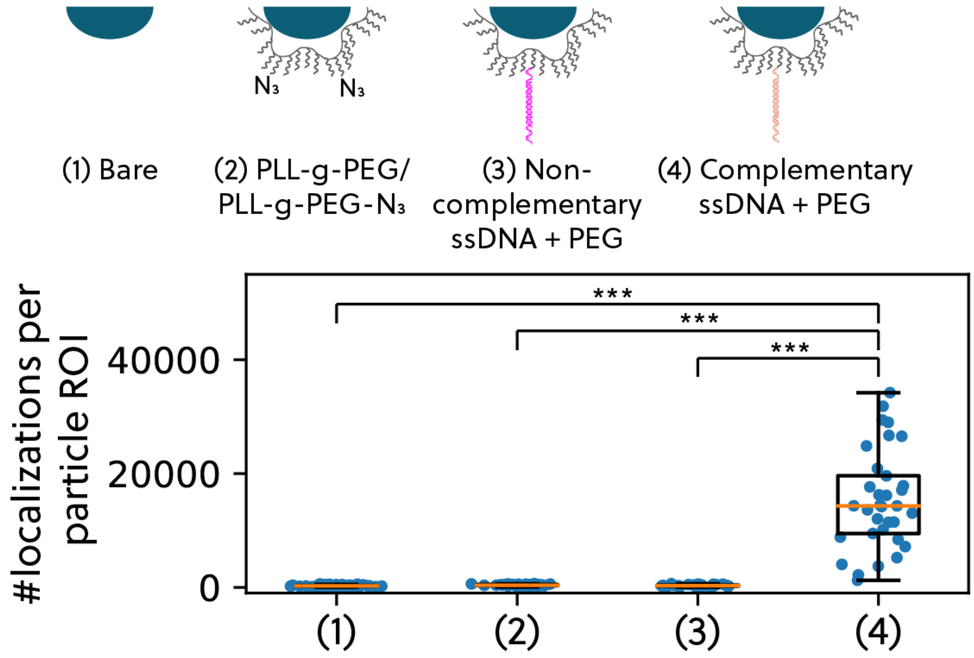

Figure S10: Control experiments for microparticles prepared with PLL-g-PEG-based click-coupling. Number of localizations per particle ROI are plotted as boxplots for different cases. Box represents the 25% to 75% percentile, whiskers 5% to 95%. Each blue filled dot represents each particle ROI. *** indicates p-values less than 0.001.

3.5 Localization uncertainty of the 3D DNA-PAINT localization data

The lateral and axial localization uncertainty for localizations obtained on an example microparticle is shown in Fig. S11. The biofunctionalized microparticle was prepared via the streptavidin-biotin coupling and incubated with 5 μM of ssDNA molecules. The median axial localization uncertainty is typically one order of magnitude larger than the median lateral localization uncertainty.

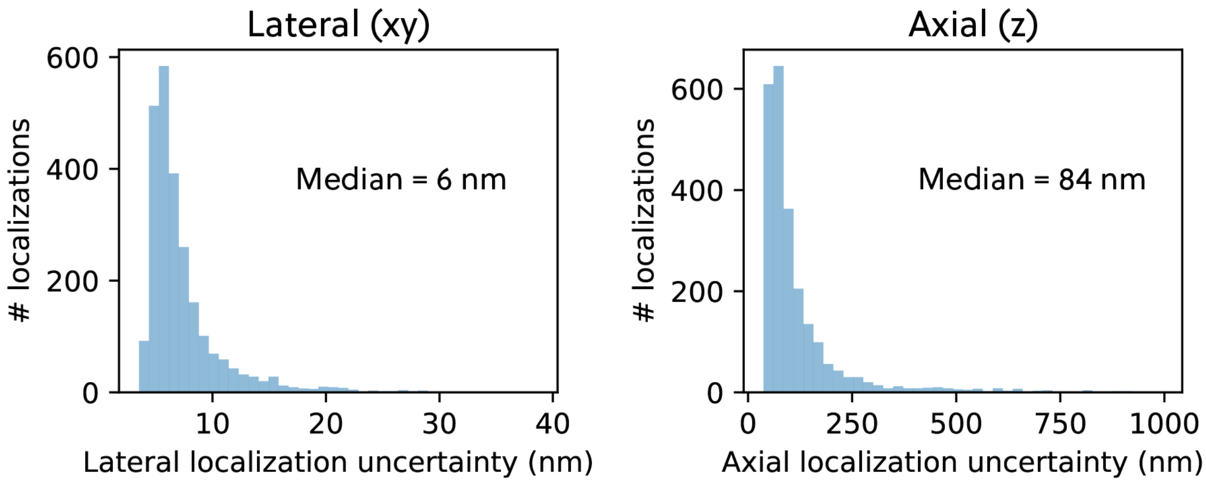

Figure S11: Localization uncertainty in the xy-direction (left panel) and z-direction (right panel) for a microparticle is shown. The median axial localization uncertainty is one order of magnitude larger than the median lateral localization uncertainty.

3.6 Molecular sampling ratio in experimental data

From the analysis developed in this work, we directly counted the number of binding events per microparticle (hence the number of observed ssDNA molecules) and compensated for the under-counting of molecules using the BiSC method to obtain the total number ssDNA molecules per microparticle. By comparing the two values, we gained insights on the percentage of ssDNA molecules that were probed during the 3D DNA-PAINT imaging. We found that approximately 73% of the ssDNA molecules per microparticle were probed during the 3D DNA-PAINT imaging, see Fig. S12.

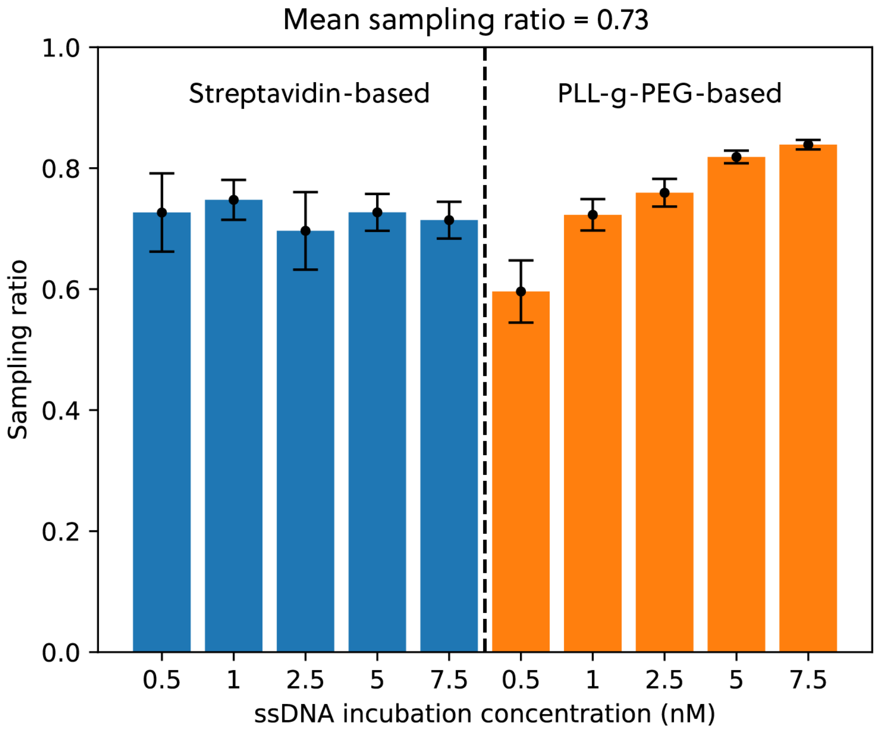

Figure S12: The percentage of ssDNA molecules probed using the 3D DNA-PAINT imaging is approximately 73%. The ratio is calculated by computing the ratio between the DC-quantified number of ssDNA molecules per particle and the BiSC-quantified value.

3.7 Streptavidin-biotin coupling: Test of hypotheses

3.7.1 Effect of pre-sonication

We hypothesized that the low number of ssDNA molecules per microparticle observed originates from the clustering of streptavidin-coated microparticles prior to ssDNA conjugation. We tested the hypothesis by pre-sonicating the microparticles prior to ssDNA conjugation. The microparticles were incubated with 0.5 μm of ssDNA molecules. In Fig. S13, we found that there were no significant differences (p-value of 0.04) between biofunctionalized microparticles with or without the pre-sonication step. Therefore, this hypothesis is rejected.

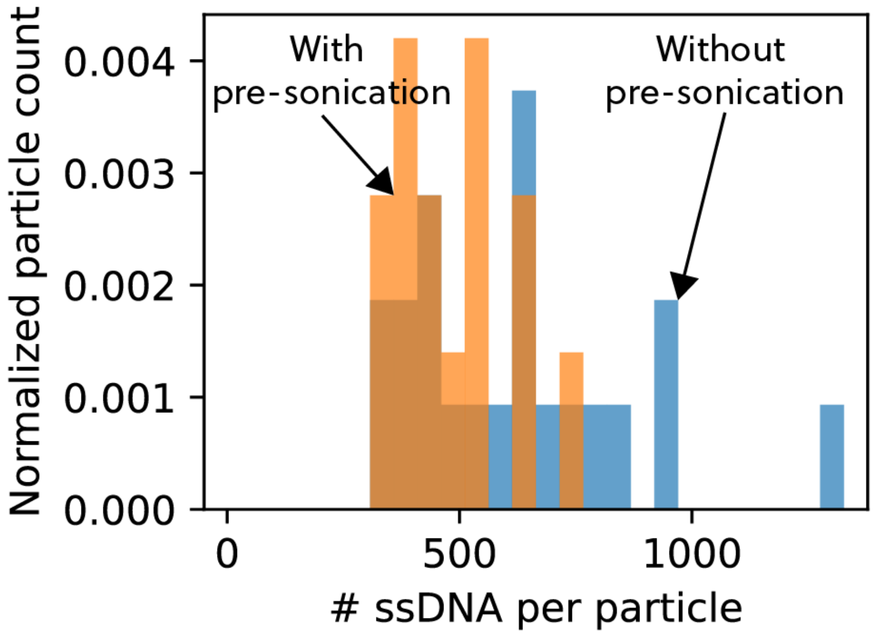

Figure S13: Effect of pre-sonication of microparticles prior to ssDNA conjugation on the number of ssDNA molecules per particle.

3.7.2 Effect of aging on the conjugation capability

In our experiments, we found a huge discrepancy between the quantified number of ssDNA molecules per microparticle and the expected value for the streptavidin-coated microparticles (three orders of magnitude lower). We suspect that the discrepancy arises from the loss of binding capacity of the microparticles due to long storage (the results shown in Fig. 5 in the main text were prepared from particle stock that has been used for multiple months). To investigate this hypothesis, we repeated the experiment using a freshly bought particle stock.

Fig. S14 showed that the number of ssDNA molecules per microparticle prepared from the fresh stock was found to be significantly different from that prepared from the aged stock. The number of ssDNA molecules per microparticle for the fresh particle stock were quantified up to low 10^3 molecules per particle, which is slightly higher than that for the aged particle stock (high 10^2 molecules per particle), indicating that particle aging has a slight influence on the binding capacity of the microparticles. However, large discrepancy still exists between the quantified value (from the fresh particle stock) and the expected value. Furthermore, we observed an interesting trend for the number of ssDNA molecules per particle for the fresh particle stock, that was previously not found in the aged particle stock. There was a slight decrease in number of ssDNA molecules per particle with increasing ssDNA incubation concentration. The decrease in quantified value may be the implication of a decrease in accessibility of the conjugated ssDNA molecules (due to an increase in conjugated ssDNA molecules on the microparticles). While the molecular origins of these observations remain unclear and to be verified, this was not investigated further as it is out of the scope of the study presented in this article.

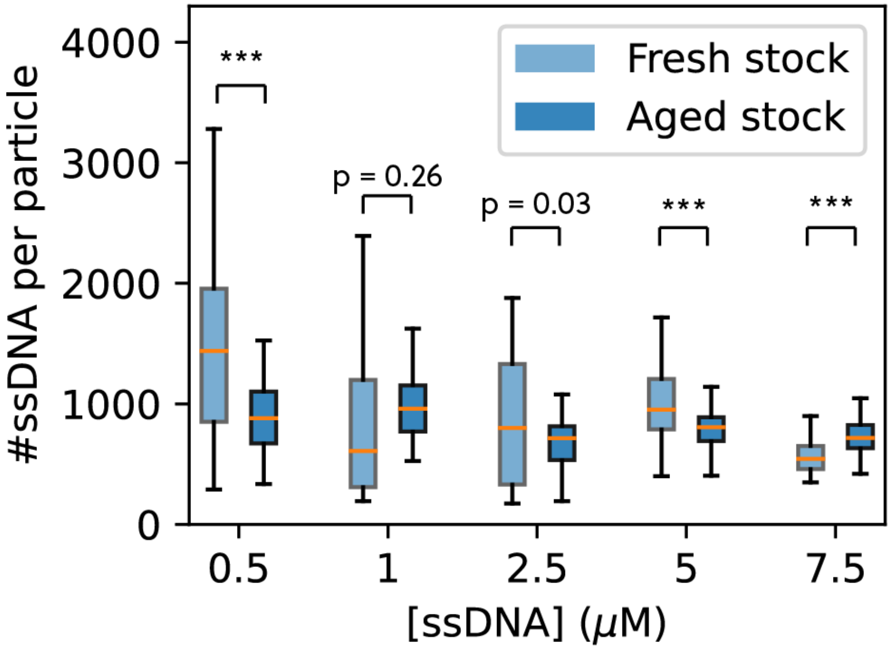

Figure S14: Two particle stocks, one of which has been used for multiple months, were compared. For some ssDNA incubation concentrations, the analysis showed significant difference between the fresh and aged particle stock. Box represents the 25% to 75% percentile, whiskers 5% to 95%. *** indicates p-value less than 0.001.

3.8 Comparison of distribution score for particles with similar distribution of number of ssDNA molecules per particle

As the number of ssDNA per microparticle affects the quantified distribution score of the microparticle, the microparticles with similar number of ssDNA molecules per particle (prepared via different coupling strategies) were compared. In Fig. S15, we showed that there is no significant difference in the number of ssDNA per microparticle between the datasets shown in Fig. 5.

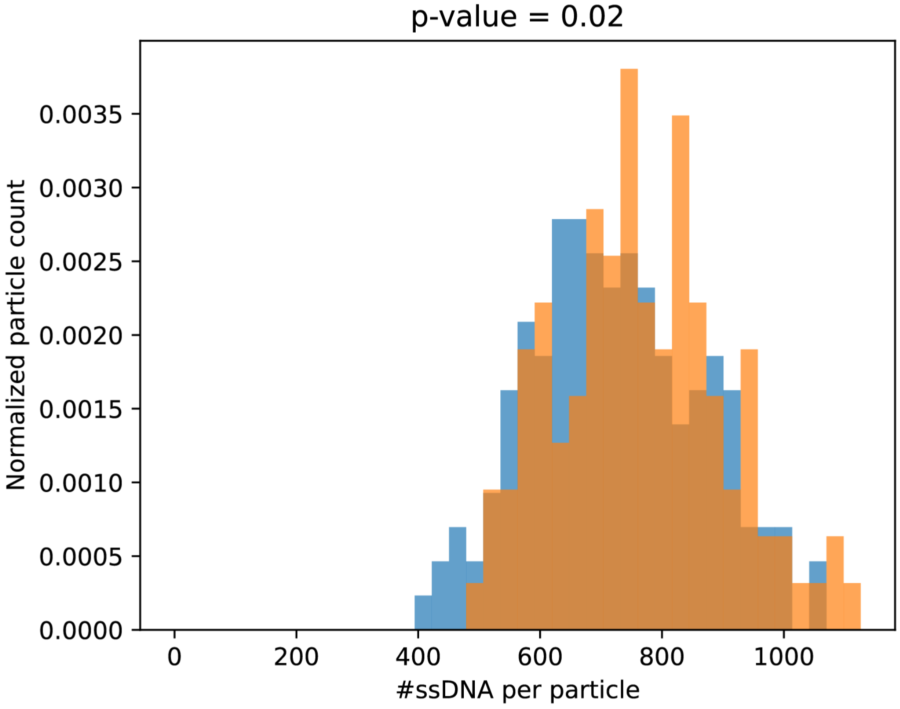

Figure S15: The datasets that were used as comparison for distribution analysis in Fig. 5 have similar distribution of number of ssDNA per microparticle. The number of ssDNA per particle is quantified using the BiSC analysis. Welch's t-test indicates no statistically significant difference in the number of ssDNA molecules per microparticle between the two datasets.

3.9 Relation between number of ssDNA molecule per particle and distribution score

In this study, we hypothesize that the ssDNA molecules coupled to the microparticles are inherently clustered due to the presence of intermediate molecules (streptavidin/PLL-g-PEG) with multiple reactive moieties per molecule. The experimental data verified this hypothesis and showed an increase in clustering degree with increasing number of ssDNA per particle, see Fig. S16.

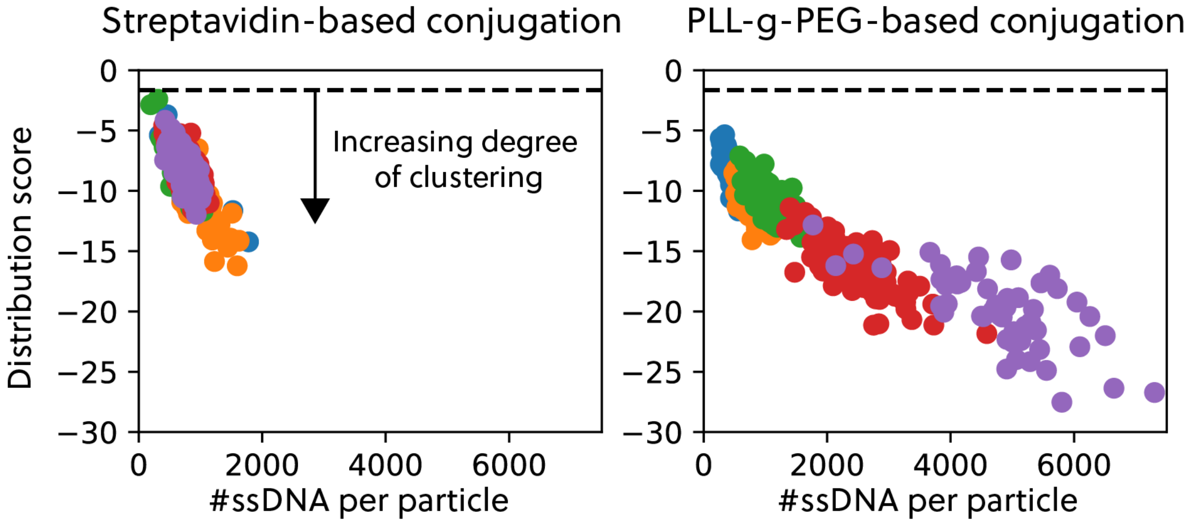

Figure S16: The relation between number of ssDNA molecules per particle and the distribution score is shown for both streptavidin-based (left panel) and PLL-g-PEG-based conjugation (right panel). The higher the number of ssDNA molecules conjugated to each microparticle, the more clustered the conjugated ssDNA molecules are. The color represents microparticles functionalized with varying ssDNA incubation concentration, while each data point represents a single microparticle.

3.10 Visual representation of molecular clustering with respect to distribution score

To illustrate how molecular clustering varies across different distribution scores, we simulated the spatial positions of coupled biomolecules. As clustering increases, the distribution scores become more negative and the nearest neighbor great-circle distances (NNDs) decrease, indicating a more compact spatial organization. These visual representations are intended to aid interpretation of the quantified distribution scores and NNDs.

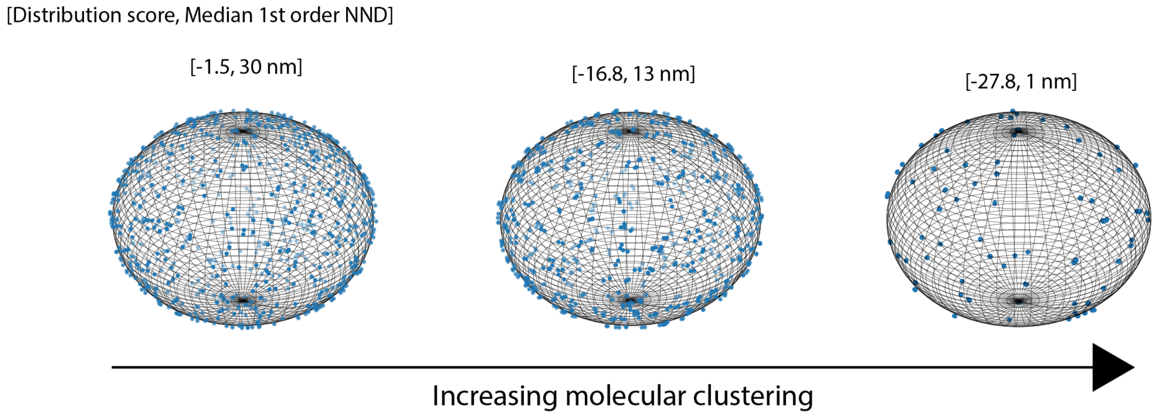

Figure S17: With increasing molecular clustering, the distribution scores become more negative, and the nearest neighbor great-circle distances (NNDs) become shorter, reflecting a more compact spatial arrangement of molecules. The number of biomolecule simulated per particle is 750.

3.11 Simultaneous detection of localizations in a particle ROI

In the experimental data, a maximum of two localizations per frame were detected within each particle ROI. Four representative frame snapshots from the same particle shown in Fig. 3(C) are provided in Fig. S18, with detected localizations marked by red crosses. While ThunderSTORM localizations are typically used directly in custom Python scripts for density and distribution analysis, here we visualized them using ThunderSTORM's built-in tools to illustrate the formation of a particle localization cloud.[S5] The average shifted histogram encodes localization density as intensity, whereas the scatter plot shows only the presence or absence of localizations per pixel. Both visualizations confirm the emergence of a well-defined particle localization cloud.

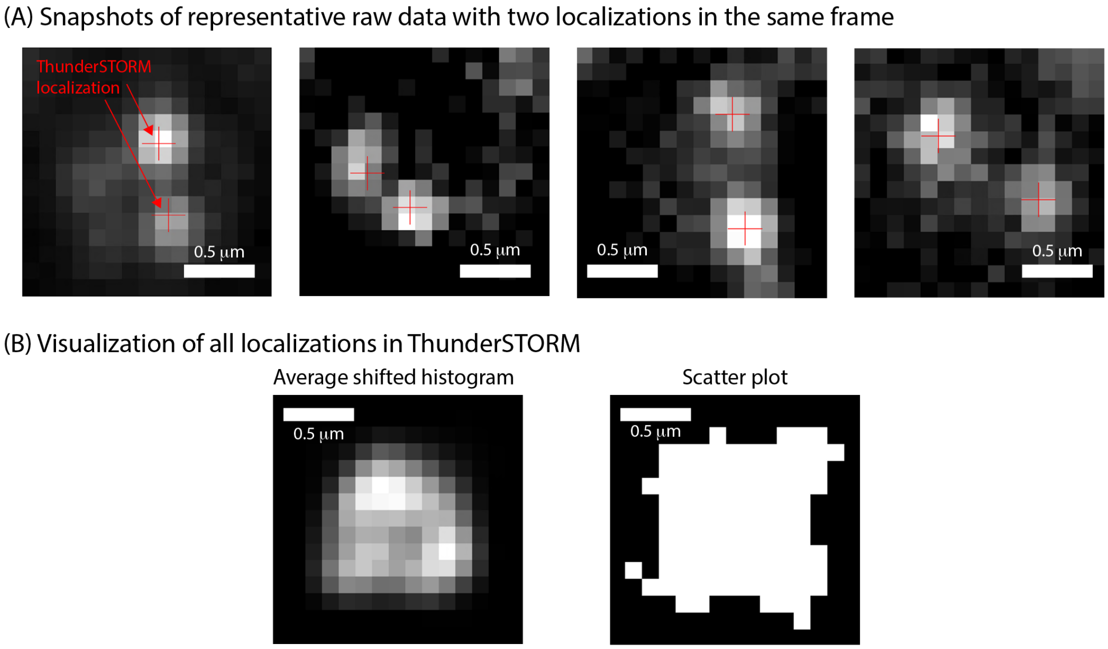

Figure S18: (A) Four representative frame snapshots of a single particle (same particle as shown in Fig. 3(C)). In each frame, two localizations (indicated by red crosses) were detected using ThunderSTORM. (B) All localizations detected over the full measurement duration (18,000 frames) are visualized using ThunderSTORM's built-in visualization tools.[S5] The average shifted histogram represents the spatial distribution of localizations, encoding localization density as intensity. In contrast, the scatter plot represents each pixel as either 0 (dark, no localization detected) or 1 (bright, localization detected), without conveying localization density. Scale bar: 500 nm.

References

(S1) Tan, W. S.; De Jong, A. M.; Prins, M. W. J. Revealing Spatial Molecular Heterogeneity of High-density Biofunctionalized Surfaces Using DNA-PAINT. *ACS Applied Materials & Interfaces* **2024**, 58191–58202.

(S2) Ester, M.; Kriegel, H.-P.; Sander, J.; Xu, X. *A Density-Based Algorithm for Discovering Clusters in Large Spatial Databases with Noise*; 1996.

(S3) Schubert, E.; Sander, J.; Ester, M.; Kriegel, H. P.; Xu, X. DBSCAN Revisited, Revisited. *ACM Transactions on Database Systems* **2017**, *42*, 1–21.

(S4) Clark, P. J.; Evans, F. C. Distance to Nearest Neighbor as a Measure of Spatial Relationships in Populations. *Ecology* **1954**, *35*, 445–453.

(S5) Ovesný, M.; Křížek, P.; Borkovec, J.; Švindrych, Z.; Hagen, G. M. ThunderSTORM: a comprehensive ImageJ plug-in for PALM and STORM data analysis and super-resolution imaging. *Bioinformatics (Oxford. Print)* **2014**, *30*, 2389–2390.
